# Amphiphilic Anionic Oligomer-Stabilized Calcium Phosphate Nanoparticles with Prospects in siRNA Delivery via Convection-Enhanced Delivery

**DOI:** 10.3390/pharmaceutics14020326

**Published:** 2022-01-29

**Authors:** Franziska Mitrach, Maximilian Schmid, Magali Toussaint, Sladjana Dukic-Stefanovic, Winnie Deuther-Conrad, Heike Franke, Alexander Ewe, Achim Aigner, Christian Wölk, Peter Brust, Michael C. Hacker, Michaela Schulz-Siegmund

**Affiliations:** 1 Institute of Pharmacy, Pharmaceutical Technology, Medical Faculty, University of Leipzig, Eilenburger Straße 15A, 04317 Leipzig, Germany; franziska.mitrach@medizin.uni-leipzig.de (F.M.); maximilian.schmid@medizin.uni-leipzig.de (M.S.); christian.woelk@medizin.uni-leipzig.de (C.W.); 2 Department of Neuroradiopharmaceuticals, Helmholtz-Zentrum Dresden-Rossendorf, Permoserstraße 15, 04318 Leipzig, Germany; m.toussaint@hzdr.de (M.T.); s.dukic-stefanovic@hzdr.de (S.D.-S.); w.deuther-conrad@hzdr.de (W.D.-C.); p.brust@hzdr.de (P.B.); 3 Rudolf-Boehm-Institute for Pharmacology and Toxicology, Faculty of Medicine, University of Leipzig, 04107 Leipzig, Germany; heike.franke@medizin.uni-leipzig.de; 4 Rudolf-Boehm-Institute for Pharmacology and Toxicology, Clinical Pharmacology, Faculty of Medicine, University of Leipzig, 04107 Leipzig, Germany; alexander.ewe@medizin.uni-leipzig.de (A.E.); achim.aigner@medizin.uni-leipzig.de (A.A.); 5 Institute of Pharmaceutics and Biopharmaceutics, Heinrich-Heine-University Düsseldorf, Universitätsstraße 1, 40225 Düsseldorf, Germany

**Keywords:** calcium phosphate nanoparticles, PEGylated terpolymer, particle characterization, siRNA delivery, cell transfection

## Abstract

Convection-enhanced delivery (CED) has been introduced as a concept in cancer treatment to generate high local concentrations of anticancer therapeutics and overcome the limited diffusional distribution, e.g., in the brain. RNA interference provides interesting therapeutic options to fight cancer cells but requires nanoparticulate (NP) carriers with a size below 100 nm as well as a low zeta potential for CED application. In this study, we investigated calcium phosphate NPs (CaP-NPs) as siRNA carriers for CED application. Since CaP-NPs tend to aggregate, we introduced a new terpolymer (o14PEGMA(1:1:2.5) NH_3_) for stabilization of CaP-NPs intended for delivery of siRNA to brain cancer cells. This small terpolymer provides PEG chains for steric stabilization, and a fat alcohol to improve interfacial activity, as well as maleic anhydrides that allow for both labeling and high affinity to Ca(II) in the hydrolyzed state. In a systematic approach, we varied the Ca/P ratio as well as the terpolymer concentration and successfully stabilized NPs with the desired properties. Labeling of the terpolymer with the fluorescent dye Cy5 revealed the terpolymer’s high affinity to CaP. Importantly, we also determined a high efficiency of siRNA binding to the NPs that caused very effective survivin siRNA silencing in F98 rat brain cancer cells. Cytotoxicity investigations with a standard cell line resulted in minor and transient effects; no adverse effects were observed in organotypic brain slice cultures. However, more specific cytotoxicity investigations are required. This study provides a systematic and mechanistic analysis characterizing the effects of the first oligomer of a new class of stabilizers for siRNA-loaded CaP-NPs.

## 1. Introduction

In recent decades, nanoparticulate systems have become an important issue as a drug delivery system, especially in cancer treatment [1].

One possible strategy to deliver nanotherapeutics is direct infusion of pharmaceuticals in the target tissue via convection-enhanced delivery (CED). CED allows for the distribution of therapeutics in normal and tumor tissue via an external pressure gradient that provides fluid convection [2,3]. Compared to diffusion, CED has been shown to result in larger distribution volumes [3]. Further benefits of CED include high local drug concentrations in the tumor areas, avoiding adverse systemic effects [3].

A promising class of therapeutics for a targeted, localized tumor application is siRNAs, which can decrease the expression of cancer-relevant genes and, in consequence, induce apoptosis in the tumor cells [4,5,6,7]. Several studies described the potential of survivin as a single molecular target in cancer cell therapy based on RNA interference without the need of additional cytotoxic factors [4,8,9,10]. However, due to the high negative charge of nucleic acids, their large molecular weight and their susceptibility to enzymatic (RNases) degradation, siRNA delivery systems are necessary to enable protection and efficient cellular uptake [11,12,13]. A generally nontoxic and naturally occurring material that can be used for gene delivery is calcium phosphate (CaP), which was developed as a transfection agent for plasmid DNA in the early 1970s by Graham and van der Eb [14,15,16]. In contrast to other gene delivery systems, the susceptibility of inorganic CaP to acid environments facilitates dissolution in endosomes and siRNA release into the cytoplasm [17]. Although this susceptibility limits a potential application in solid tumors, application of siRNA-loaded CaP-NPs after tumor resection might help to inhibit relapse.

CaP particles tend to aggregate after their preparation and require the addition of a suitable stabilizer for a successful in vivo application [18,19]. Different strategies for stabilization have already been published including PEGylated stabilizers with anionic functional groups for interaction with CaP-NPs [20,21,22,23,24,25,26], coating with lipids [27,28,29,30] and coating with nucleic acids and polyethyleneimines (PEIs), resulting in core–shell particles [31,32,33]. Darvan 821-A^®^ (R.T. Vanderbilt, Norwalk, CT, USA) represents a commercially available CaP stabilizer that is based on polyacrylate [34,35] and provides negative charges capable of binding to CaP-NPs [34,35,36].

With the objective to also employ a polyanionic substance and implement an amphiphilic design for increased interfacial affinity as well as functional groups for further chemical modifications, we focused on anhydride-containing amphiphilic oligomers to stabilize nanoparticles of a size below 100 nm. An oligomeric copolymer of maleic anhydride (MA), tetradecyl acrylate (TDA or 14 in oligomer code) and monomethoxy poly(ethylene glycol)methacrylate (PEGMAc or PEG in polymer code) combines the desired functionalities. Negative charges mediating affinity to positively charged domains of CaP during precipitation can be obtained by hydrolysis or aminolysis of anhydride groups. Working with anhydrides as reactive precursors for carboxylate groups ensures solubility in organic solvents during synthesis and subsequent chemical modification. The terpolymer further contains TDA as a lipophilic comonomer with a medium chain length to provide surface activity, and PEG950-methacrylate for steric stabilization of the nanoparticles [37,38,39]. This molecular weight was selected because several reports suggested a decreased cellular uptake of particles decorated with PEG of molecular weights above 2000. Anhydrides can also be used for convenient covalent modification with fluorescent or other diagnostic labels. To our knowledge, this is the first report on an amphiphilic anionic oligomer for CaP stabilization which is also capable of instant chemical modification.

In this study, we systematically investigated effects of our terpolymer on particle size distribution with the aim to generate siRNA-carrying NPs with a mean diameter below 100 nm for CED application. We applied zeta potential measurements to provide information on the influence of terpolymer binding on the surface charge. To investigate a potential competition between siRNA and the terpolymer, we used fluorescence labeling of both in order to discriminate between the bound and free terpolymer and siRNA.

Cytocompatibility was assessed according to ISO 10993-5 with L929 mouse fibroblasts [40], and siRNA silencing efficiency was studied in the rat glioblastoma cell line F98. Biocompatibility in intact tissue was studied in ex vivo brain tissue slice cultures. To assess the CaP-NP distribution in the brain using CED, early preliminary in vivo investigations on the NP distribution were performed.

## 2. Materials and Methods

### 2.1. Materials

CaCl_2_ × 2H_2_O, HEPES, Tris-HCl and NaCl were purchased from PanReac AppliChem ITW reagents (Darmstadt, Germany). Na_2_HPO_4_ × 2H_2_O, Dulbecco’s phosphate-buffered saline w/o Mg w/o Ca, penicillin/streptomycin, Dulbecco’s Modified Eagle Medium high glucose, horse serum, fetal bovine serum, picric acid and 3,3′-diaminobenzidine tetrahydrochloride were purchased from Sigma-Aldrich (St. Louis, MO, USA). Methoxy(PEG) methacrylate (Mn: 950, 300 ppm BHT and 100 ppm MEHQ as an inhibitor), 4,4′-azobisisobutyronitrile and trimethylamine were purchased from Sigma-Aldrich Chemie GmbH (Taufkirchen, Germany). Rotitest^®^ Vital, hematoxylin solution acc. to Gill II, propidium iodide and n-butylacetate were purchased from Carl Roth GmbH + Co. KG (Karlsruhe, Germany). Minimal essential medium, glutamine, Annexin V-FITC and Hank’s Balanced Salt Solution were purchased from Gibco/Thermo Fisher Scientific (Waltham, MA, USA).

Lipofectamine^TM^ RNAiMAX, High-Capacity cDNA Reverse Transcription Kit, TaqMan™ Universal PCR Master Mix, no AmpErase™ UNG and TaqMan™ Gene Expression Assays Rn00574012_m1 (BIRC5) and Rn03302271_gH (RPLP0) were purchased from Applied Biosystems^TM^ by Thermo Fisher Scientific (Foster City, CA, USA). RNeasy Mini Kit, RNase-free H_2_O, RNase-free DNase set, AllStars Negative Control siRNA, AllStars Negative Control siRNA labeled with AlexaFluor^TM^ 647 (AF647) and BIRC5 siRNA Rn_RGD:70499_4 were purchased from Qiagen (Hilden, Germany). BLOCK-iT™ fluorescent oligo, calcein AM and ethidium homodimer-1 were purchased from Invitrogen (Carlsbad, CA, USA). Rabbit anti-active caspase-3 antibody was purchased from MBL (Woburn, MA, USA). Biotinylated secondary antibody (immunoglobulin IgG H + L) for detection of caspase-3 antibody and ABC-Elite kit were purchased from Vector Laboratories (Burlingame, CA, USA). Paraformaldehyde, 2-methylbutane and Entellan^®^ were purchased from Merck (Darmstadt, Germany). Isoflurane was purchased from CP-Pharma Handelsgesellschaft mbH (Burgdorf, Germany).

Histoacryl was purchased from B. Braun (Melsungen, Germany). Cresyl violet was purchased from Fluka Chemie (Buchs, Switzerland). Glutaraldehyde was purchased from Serva (Heidelberg, Germany). Cyanine-5-amine was purchased from Lumiprobe (Hannover, Germany). Lactate dehydrogenase (LDH) cytotoxicity kit was purchased from Roche (Basel, Switzerland). Triton X-100 was purchased from Ferak Berlin GmbH (Berlin, Germany). Tetradecylacrylate was purchased from TCl Deutschland GmbH (Eschborn, Germany). Diethyl ether, THF and ethanol were purchased from VWR International GmbH (Darmstadt, Germany). Deuterated chloroform (CDCl3) with an internal reference of 0.03% tetramethylsilane and other deuterated NMR solvents were purchased from ARMAR (Europa) GmbH (Leipzig, Germany). Maleic anhydride and aniline were purchased from Acros Organics (Geel, Belgium). Sodium hydroxide was purchased from Th. Geyer (Renningen, Germany). The 25% ammonia hydroxide solution was purchased from Grüssing GmbH (Filsum, Germany). Caspase-Glo^®^ 3/7 assay was purchased from Promega (Madison, WI, USA).

### 2.2. Synthesis and Characterization of the Amphiphilic Terpolymer o14PEGMA(1:1:2.5)_NH_3_

The pristine terpolymer oligo(TDA-co-PEG-co-MA) (o14PEGMA)(1:1:2.5) was synthesized by free radical polymerization from tetradecylacrylate (TDA or 14 in oligomer code), methoxy poly(ethylene glycol)(900) methacrylate (PEG) and maleic anhydride (MA) in a molar ratio of 1:1:2.5, as described before for similar anhydride-containing oligomers [41,42,43,44].

In brief, the monomers were weighted and dissolved in anhydrous THF at 60 °C under nitrogen purge; polymerization was initiated by the addition of 2 mol% AIBN and left to react for 18 h. Thereafter, the reaction mixture was concentrated by rotoevaporation, redissolved in methylene chloride and precipitated in diethyl ether. The precipitation step was repeated twice, and the isolated precipitate was dried under vacuum. The pristine oligomer was converted into the corresponding ammonium salt by anhydride hydrolysis in 1 M ammonia solution at 60 °C overnight [45,46]. Excess ions were removed by dialysis against deionized water (200 mL) using a 0.5–1 kDa MWCO Float-A-lyzer dialysis device (5 mL) for 10 h and repeated changes of the outer phase [47].

The molecular weight distribution of the pristine terpolymer was analyzed by gel permeation chromatography (GPC) in THF using two columns (SDV 1000 and 100,000 Å with a 5 μm particle size, 8 m × 300 m) and one precolumn (SDV 5 μm; 8 m × 50 mm) (PSS, Mainz, Germany) relative to poly(styrene standards) using PSS Ready Cal-Kit (PSS, Mainz, Germany), as previously described [41,43].

The chemical composition of pristine terpolymers was derived from ^1^H-NMR analysis and acid–base titration. Proton NMR of sample solutions with concentrations of 0.5 mg/mL in deuterated chloroform was recorded on a 400 MHz, Bruker AVANCE III HD spectrometer (Bruker, Billerica, MA, USA) and analyzed using the MNova software version 11.0.3–18688 (Mestrelab research SL, Santiago de Compostela, Spain). The amount of incorporated PEG monomer was obtained from the proton signals between 3.42 and 3.86 ppm relative to the amount of incorporated TDA using the methyl end group at 0.75–1.00 ppm (3H). The amount of incorporated MA was determined by conductometric titration, as previously described [42]. This reference also describes the determination of chemically intact anhydrides by titration according to Brown and Fujimori.

### 2.3. Covalent Derivatization of the Oligomer with Fluorescent Dye Cy5

Prior to hydrolytic dissociation of the anhydride groups in pristine o14PEGMA, a defined amount of anhydrides can be covalently derivatized by incubation of an oligomer solution with a solution of a functional amine molecule. To immobilize a fluorescent label to the oligomer, cyanine-5-amine (Cy5-amine) in a molar amount equal to 25% of the intact anhydrides per oligomer was added to an acetonic oligomer solution. Thereafter, 5% of TEA relative to intact MA was added, and the solution was left to stir at room temperature for 4 h [22,42]. Afterwards, the remaining anhydrides were hydrolyzed, and the product was purified and isolated as described above by dialysis (MWCO: 0.1–0.5 kDa) against water.

### 2.4. Preparation of CaP-NPs

CaP-NPs were precipitated via combination of a CaCl_2_ and a Na_2_HPO_4_ solution in the presence of siRNA (co-precipitation). Two different methods were applied to produce unloaded or siRNA-loaded CaP-NPs [22].

For method 1, 25 µL of a CaCl_2_ solution (2.5 µL of 2.5 M CaCl_2_ × 2H_2_O containing 0, 1 or 10 µmol/L siRNA and 10 mmol/L Tris pH 7.0) was mixed with 25 µL of a PO_4_^3−^ solution (140 mmol/L NaCl, 1.5/3.75/6.0 mmol/L Na_2_HPO_4_ × 2H_2_O, 50 mmol/L HEPES, pH 7.0). After 4 s, 25 µL o14PEGMA(1:1:2.5)_NH_3_ (in 50 mmol/L HEPES pH 7.0) was added to the calcium phosphate solution. For production of the final CaP-NPs, the solution was gently mixed and vortexed for 2 s.

For method 2, 25 µL of a PO_4_^3−^ solution (140 mmol/L NaCl, 1.5/3.75/6.0 mmol/L Na_2_HPO_4_, 50 mmol/L HEPES, pH 7.0) was first mixed with 25 µL of o14PEGMA(1:1:2.5)_NH_3_ (in 50 mmol/L HEPES pH 7.0) solution. Then, the mixture was added to a 25 µL CaCl_2_ solution (2.5 µL CaCl_2_ containing 0, 1 or 10 µmol/L siRNA and 10 mmol/L Tris pH 7.0). For production of the final CaP-NPs, the solution was gently mixed and vortexed for 2 s. Table 1 and Table 2 give an overview of the different concentrations of the oligomer for NP stabilization as well as different siRNA amounts used for loading of oligomer-stabilized CaP-NPs and siRNA transfection.

#### Concentration of the CaP-NPs

Due to the limitation of the injection volume (5 µL) by CED, oligomer-stabilized CaP-NPs were concentrated by ultrafiltration prior to injection. CaP-NPs were prepared as described above and loaded with 10 µmol/L siRNA. After 30 min of complexation, NP solution was transferred to Amicon Ultra-4 100K centrifugal filter units (Merck Millipore, Tullagreen, Ireland) and centrifuged (Centrifuge 5430 R, Eppendorf, Hamburg, Germany) for 4 min at 2000 rcf.

### 2.5. Particle Size and Zeta Potential Measurements

Particle size measurements were performed by via Nanoparticle Tracking Analysis (NTA, NanoSight LM 10, Malvern Panalytical, Kassel, Germany) and laser diffraction analysis (Mastersizer 3000, dispersion unit: Hydro SV; Malvern Panalytical, Kassel, Germany). NTA measurements were performed at 25 °C with a measurement time of 30 s and a detection threshold of 8. Laser diffraction analysis was performed to simultaneously observe NPs and aggregates. Measurement parameters were set as follows: refractive index 1.6; absorption index: 0.001; density: 3.14 g/cm^3^; and a measurement time of 10 s per sample.

For determination of zeta potentials, we applied electrophoretic light scattering (Litesizer 500) in combination with Univette Low Volume (both Anton Paar, Graz, Austria). The zeta potential measurements were calculated using the Smoluchowski method with the following specifications: medium viscosity 0.8903 mPas, temperature 25 °C, filter optical density 3.2102 and automatic voltage adjustment.

### 2.6. Determination of the siRNA Binding Capacity

**Figure 1:**
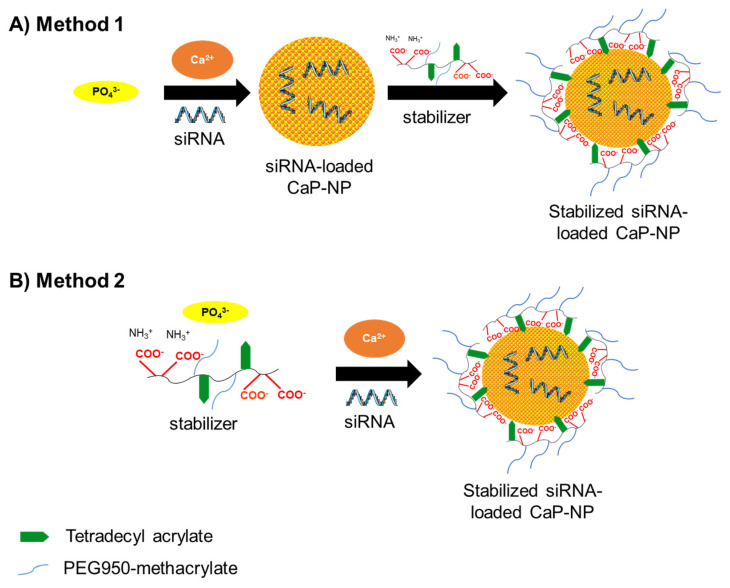
Schematic illustration o14PEGMA(1:1:2.5)_NH_3_ CaP-NP preparation using two different methods. (**A**) In method 1, a stabilizer was added 4 s after the CaP precipitation. (**B**) In method 2, a solution of a stabilizer and PO_4_^3−^ was mixed with a siRNA-containing Ca^2+^ solution.

The siRNA binding capacity of different CaP-NP formulations was evaluated by determining the proportion of unbound siRNA. To this end, FITC-labeled siRNA was used. The CaP-NPs were prepared according to methods 1 and 2 (Figure 1) and loaded with 1 µmol/L FITC-siRNA. After preparation, unbound siRNA was separated from CaP-NPs using centrifugal filter units (Amicon Ultra-4 100K, Merck Millipore, Tullagreen, Ireland) at 2000 rcf for 4 min. After centrifugation, the fluorescence intensities (relative fluorescence units, RFU) (excitation: 494 nm, emission: 519 nm) of the flow-throughs were measured in a plate reader (Synergy H1, BioTek, Bad Friedrichshall, Germany). The proportion of unbound FITC-siRNA [%] was calculated according to the following equation, where the control represents 1 µmol/L FITC-siRNA without CaP-NPs:

$$\mathrm{unbound}\;\mathrm{FITC}-\mathrm{siRNA}\;\left[\%\right]=(\frac{{\mathrm{RFU}}_{\mathrm{flow}-\mathrm{through}\;\mathrm{sample}}}{{\mathrm{RFU}}_{\mathrm{flow}-\mathrm{through}\;\mathrm{control}}})\;\times \;100$$

### 2.7. Determination of the Oligomer Binding Capacity

The binding capacity of the oligomer to CaP-NPs was analyzed by using o14PEGMA(1:1:2.5)_NH_3_ labeled with Cy5-amine. Oligomer-stabilized CaP-NPs were prepared according to method 2 (Figure 1B) and loaded with 1 µmol/L BIRC5 siRNA. After 30 min of incubation, CaP-NP solutions were loaded on centrifugal filters and processed as described in Section 2.6. For excluding binding of the stabilizer to centrifugal filter units, we applied a cyanine-5-amine-labeled stabilizer without CaP-NPs as a control. We performed the same ultrafiltration procedure as for the CaP-NP groups to obtain a small volume of retentate for fluorescence measurements. Fluorescence intensities (excitation: 650 nm, emission: 676 nm) of the retentate (stabilizer bound to CaP-NPs) and flow-through (unbound stabilizer) were determined. The proportion of oligomer [%] in the retentate and flow-through was calculated according to the following equation:

$$\mathrm{oligomer}\;\left[\%\right]=\left(\frac{{\mathrm{RFU}}_{\mathrm{sample}\;(\mathrm{flow}-\mathrm{through}\;\mathrm{or}\;\mathrm{retentate})}}{{\mathrm{RFU}\;\mathrm{control}}_{\mathrm{retentate}}{+\mathrm{RFU}\;\mathrm{control}}_{\mathrm{flow}-\mathrm{through}}}\right)\;\times \;100$$

### 2.8. Cell Culture

F98 cells (ATCC CRL-2397, Manassas, VA, USA) were cultivated in Dulbecco’s Modified Eagle Medium high glucose supplemented with 10% FBS and 1% penicillin/streptomycin in a humified atmosphere of 5% CO_2_ at 37 °C. Every 2–3 days, subcultivation was conducted. Before harvesting cells for in vitro experiments, 70–80% confluence of cells was obtained.

For biocompatibility testing, L929 cells (Cell Lines Service, Eppelheim, Germany) were cultivated in Dulbecco’s Modified Eagle Medium low glucose supplemented with 10% FBS and 1% penicillin/streptomycin in a humified atmosphere of 5% CO_2_ at 37 °C and subcultured as described for F98 cells.

### 2.9. Cellular Uptake of CaP-NPs

The cellular uptake of CaP-NPs in F98 cells was analyzed by using AllStars Negative Control siRNA AF647 in the rat glioblastoma cell line F98. The day before transfection, F98 cells were seeded with a density of 30,000 cells/well into 48-well plates (Costar^®^ 48-well, Corning, NY, USA) and cultivated under standard conditions (37 °C, 5% CO_2_). On the day of transfection, cell culture medium was removed and replaced with 300 µL DMEM high glucose supplemented with 2% FBS without antibiotics. CaP-NPs were prepared as described above with 3.75 mmol/L PO_4_^3−^ and 40 µmol/L o14PEGMA(1:1:2.5)_NH_3_. As siRNA loading amounts, 1 and 10 µmol/L were used. Fluorescence signals were analyzed at 4 and 24 h after addition of CaP-NPs (75 µL) by using a Nikon Eclipse TE2000-S Inverted Microscope (Nikon Corp., Tokyo, Japan) with the fluorescent lamp C-HGFI (Nikon Corp., Tokyo, Japan). Before analysis, cells were washed twice with 1× PBS to avoid quenching of fluorescence signals by CaP-NPs on the cell monolayer. To obtain the 24 h values, cells were washed twice with 1× PBS and cultured in DMEM high glucose + 2% FBS + 1% penicillin/streptomycin under standard conditions (5% CO_2_, 37 °C) until analysis.

### 2.10. BIRC5 siRNA Transfection Experiments

The siRNA transfection efficiency of CaP-NPs was assessed in the rat glioblastoma cell line F98. As a molecular target, BIRC5, encoding the inhibitor of apoptosis protein survivin, was used. Twenty-four hours before transfection, F98 cells were seeded with a density of 30,000 cells/well into 48-well plates (Costar^®^ 48-well, Corning, NY, USA) and cultivated under standard conditions (37 °C, 5% CO_2_). On the day of transfection, cell culture medium was removed and replaced with 300 µL DMEM high glucose supplemented with 2% FBS without antibiotics. CaP-NPs were prepared as described above according to method 2 with 40 µmol/L oligomer (Figure 1) and loaded with 1 µmol/L BIRC5 siRNA (sense strand: 5′-CUAUUGUGACCUGGACUUATT-3′; antisense strand: 5′-UAAGUCCAGGUCACAAUAGAG-3′). As a non-coding control siRNA, we used AllStars Negative Control siRNA (sense strand: 5′-proprietary-3′; antisense strand: 5′-proprietary-3′) from Qiagen (Hilden, Germany).

CaP-NPs were prepared with 40 µmol/L oligomer according to method 2. In total, 75 µL CaP-NP solution was added per well of a 48-well plate (Costar^®^ 48-well, Corning, NY, USA).

To determine normal BIRC5 gene expression levels as well as unaffected cell viabilities, untreated F98 cells served as control. This control group was neither treated with CaP-NPs nor with the naked siRNA. As further transfection controls, we applied 1 µmol/L of the respective naked siRNA (BIRC5/non-coding) to exclude any effect of siRNA in the absence of the gene delivery system. Unloaded CaP-NPs as well as CaP-NPs loaded with non-coding siRNA served as further controls.

After 5 h of incubation at 3% CO_2_, cells were washed twice with 1 × PBS. Afterwards, DMEM high glucose supplemented with 2% FBS and 1% penicillin/streptomycin was added, and cells were cultivated until analysis under standard conditions (37 °C, 5% CO_2_).

#### 2.10.1. Determination of the BIRC5 Gene Expression Levels

BIRC5 gene expression levels were analyzed with quantitative real-time PCR using BioRad CFX Touch (BioRad, Feldkirchen, Germany). For this, F98 cells were transfected as described above. On day 2 post-transfection, cells were harvested, and total RNA was extracted by using the RNeasy Mini Kit in combination with a DNase digestion using RNase-free DNase set according to the manufacturer’s protocols. Reverse transcription was performed using the High-Capacity cDNA Reverse Transcription Kit according to the manufacturer’s instructions. Real-time PCR was performed using the TaqMan gene expression assay in combination with TaqMan™ Master Mix (primer sequences for BIRC5: Rn00574012_m1). BIRC5 gene expression levels were normalized to the housekeeping gene RPLP0 (Rn03302271_gH) using the 2^(−ΔΔCT)^ method. Unloaded CaP-NPs and non-coding siRNA-loaded CaP-NPs, as well as untreated F98 cells and F98 cells treated with naked siRNA, served as controls.

#### 2.10.2. Determination of the Cell Viability

The effect of reduced BIRC5 gene expression levels on cell viability was analyzed with the WST-8 assay (Rotitest^®^ Vital) based on the formation of formazan by viable cells only. To this end, F98 cells were transfected as described above. On day 3 post-transfection, cell culture medium was removed and replaced with 300 µL fresh culture medium per well of a 48-well plate (Costar^®^ 48-well, Corning, NY, USA). Immediately thereafter, 30 µL of Rotitest^®^ Vital reagent was added per well of a 48-well plate (Costar^®^ 48-well, Corning, NY, USA) and cells were incubated for 1 h at 37 °C and 5% CO_2_. Cell culture supernatants were collected, and formazan absorbance was measured at 450 nm using a plate reader (Synergy H1, Biotek, Bad Friedrichshall, Germany). The cell viability [%] was calculated according to the following equation, where the untreated control represents untreated F98 cells:

$$\mathrm{cell}\;\mathrm{viability}\;\left[\%\right]=\left(\frac{{\mathrm{OD}}_{450\;\mathrm{nm}}\mathrm{Sample}}{{\mathrm{OD}}_{450\;\mathrm{nm}}\mathrm{untreated}\;\mathrm{cells}}\right)\;\times \;100$$

#### 2.10.3. Caspase Assay and Annexin V Staining

For Annexin V-FITC/propidium iodide (PI) staining, F98 rat glioblastoma cells were seeded in 48-well plate (Costar^®^ 48-well, Corning, NY, USA) and transfected as described above. On day 2 post-transfection, cells were washed twice with 1× PBS and trypsinized, and cell culture medium was added to stop the trypsinization. Subsequently, cells were collected and centrifuged (Biofuge Pico, Heraeus, Hanau, Germany) at 1200 rpm for 5 min. The cell pellets were washed with 500 µL PBS and again centrifuged as above. Finally, the cells were resuspended in 100 µL 1× Annexin binding buffer (10 mM HEPES, 140 mM NaCl and 2.5 mM CaCl_2_, pH 7.4) and stained with 2.5 µL Annexin V-FITC as well as 2 µL PI solution (2 mg/mL). After 15 min of incubation in the dark at room temperature, the samples were filled up with an additional 400 µL 1× Annexin binding buffer and analyzed using an Attune^®^ Acoustic Focusing Cytometer (Thermo Fisher Scientific, Schwerte, Germany). For analysis of apoptosis, Annexin-positive cells in the upper and lower right quadrants were considered.

For the quantification of caspase-3/7 activity, the Caspase-Glo^®^ 3/7 assay was used. F98 rat glioblastoma cells were seeded in 48 wells (Costar^®^ 48-well, Corning, NY, USA) and transfected with CaP-NPs as described above. On day 3 post-transfection, the Caspase-Glo^®^ 3/7 assay was performed according to the manufacturer’s protocol. After 1 h of incubation at room temperature in the dark, 200 µL was transferred to a 96-well plate (Costar^®^ 96-well, Corning, NY, USA). Luminescence was measured by using a Fluostar Optima Reader (BMG Labtec, Ortenberg, Germany). To normalize different cell densities caused by BIRC5 silencing (reduced cell proliferation), the WST-8 assay was performed in parallel for determination of the luminescence signal/WST-8 signal ratio. For this, 30 µL Rotitest^®^ Vital reagent was added per well of a 48-well plate (Costar^®^ 48-well, Corning, NY, USA) and cells were incubated for 1 h at 37 °C and 5% CO_2_. Cell culture supernatants were collected, and formazan absorbance was measured at 450 nm using a plate reader (Synergy H1, Biotek, Bad Friedrichshall, Germany). Data were normalized to untreated F98 cells.

### 2.11. In Vitro Cytocompatibility Testing

The cytocompatibility testing of o14PEGMA(1:1:2.5)_NH3-stabilized CaP-NPs was performed according to ISO 10993-5 [40] using L929 mouse fibroblasts (Cell Lines Service, Eppelheim, Germany) at passages 38 and 39. L929 fibroblasts were seeded at a density of 30,000 cells/well in a 48-well plate (Costar^®^ 48-well, Corning, NY, USA) (non-concentrated CaP-NPs) or 10,000 cells/well in a 96-well plate (Costar^®^ 96-well, Corning, NY, USA). After 24 h, cell culture medium was removed and replaced with DMEM low glucose supplemented with 10% FBS without antibiotics.

CaP-NPs were prepared as described above according to method 2 with 40 µmol/L oligomer, loaded with 0 or 1 µmol/L siRNA and 75 µL non-concentrated CaP-NPs added per well of a 48-well plate (Costar^®^ 48-well, Corning, NY, USA). For the concentrated group, CaP-NPs were centrifuged (Centrifuge 5430 R, Eppendorf, Hamburg, Germany) for 4 min at 2000 rcf using Amicon Ultra-4 100K centrifugal filters (Merck Millipore, Tullagreen, Ireland). After concentration, 5 µL CaP-NPs was added per well of a 96-well plate (Costar^®^ 96-well, Corning, NY, USA). Untreated L929 cells served as a negative cytotoxicity control. As a positive toxicity control, L929 cells were treated with 70% (*v*/*v*) ethanol in water.

After 24 h, media were exchanged with fresh culture medium in all groups, and the cells were kept for another 48 h at 37 °C and 5% CO_2_. Analysis was conducted after 24 and 72 h using the Live/Dead Assay and WST-8 assay.

#### 2.11.1. Determination of the Metabolic Activity

The WST-8 assay (Rotitest^®^ Vital) was performed according to the manufacturer’s protocol to analyze the metabolic activity. The metabolic activity [%] was calculated according to the following equation:

$$\mathrm{metabolic}\;\mathrm{activity}\;\left[\%\right]=\left(\frac{{\mathrm{OD}}_{450\;\mathrm{nm}}\mathrm{sample}}{{\mathrm{OD}}_{450\;\mathrm{nm}}\mathrm{untreated}\;L929\;\mathrm{cells}}\right)\;\times \;100$$

#### 2.11.2. Live/Dead Assay

For the Live/Dead assay, cells were washed with PBS and incubated with a mixture of calcein AM (2 µM) and ethidium homodimer-1 (4 µM) in DMEM low glucose w/o phenol red supplemented with 10% heat-inactivated FBS for 45 min at 37 °C and 5% CO_2_. Cells were then washed twice with PBS to remove extra dye and analyzed through fluorescent microscopy using a Nikon Eclipse TE2000-S Inverted Microscope (Nikon Corp., Tokyo, Japan) and the fluorescent lamp C-HGFI (Nikon Corp., Tokyo, Japan). The results were reported as fluorescent images showing red (dead) and green (living) cells.

### 2.12. Tissue Slice Cultures

#### 2.12.1. Preparation and Treatment

Athymic nude mice (NMRI Nude Mouse; Crl:NMRI-Foxn1nu, Charles River Laboratories, Sulzfeld, Germany) were kept under standard conditions strictly according to the national regulations of animal welfare, with access to lab food and water ad libitum. This project did not involve animal experiments.

Striatal brain tissue slices were prepared from ~4-month-old mice and cultivated as previously described [48,49]. In brief, immediately after mouse sacrifice, the brain was carefully removed under aseptic conditions and placed into a Petri dish containing warm 1.5% agarose solution. After solidification on ice, a block containing the striatum was prepared and glued onto the vibratome specimen holder using Histoacryl. Coronal 300 µm sections were cut using a Leica VT1200S Vibratome (Leica Microsystems, Nussloch, Germany) and collected in ice-cold preparation medium (Minimal essential medium, 2 mmol/L glutamine, 50 µg/mL gentamicin (pH 7.3)). For air–liquid interface culture, 6-well tissue culture (TC) plates (Sarstedt, Nümbrecht, Germany) and TC inserts with a semi-permeable porous membrane (Sarstedt, Nümbrecht, Germany) were used. The striatal tissue slices were transferred onto the membranes, and the inserts were placed in 1 mL incubation medium per well (50% Minimal essential medium, 25% Hank’s Balanced Salt Solution, 25% heat-inactivated horse serum, 2 mmol/L glutamine, 0.625% glucose; pH 7.2) containing 50 µg/mL gentamicin per well. After 15 min, 5 µL concentrated CaP-NPs (see below) or medium alone was directly pipetted onto the top of the slices. The siRNA-loaded CaP-NPs were prepared according to method 2 using 3.75 mmol/L Na_2_HPO_4_ and loaded with 1 µmol/L siRNA as described before. For stabilization, we added 40 µmol/L terpolymer. In total, we applied three different groups: medium control, non-concentrated CaP-NPs and concentrated CaP-NPs. Concentration of CaP-NPs was conducted by using Amicon Ultra-4 100K centrifugal filter units (Merck Millipore, Tullagreen, Ireland) as described before. The striatal tissue slice cultures were incubated under standard cell culture conditions (humidified atmosphere, 37 °C, 5% CO_2_) for three days, unless stated otherwise, prior to collecting the incubation media for LDH release assays and analysis of the tissues. The tissue slices were fixed in a solution containing 4% paraformaldehyde, 0.1% glutaraldehyde and 0.2% picric acid in 100 mmol/L Sorensen’s phosphate buffer (SPB, pH 7.35) for 2 h, prior to thorough washing and storage at 4 °C. Using the vibratome (Leica VT1200S Vibratome, Leica Microsystems, Nussloch, Germany), free-floating 50 µm slices were prepared and collected in 0.1 M TBS (pH 7.6).

#### 2.12.2. Microscopic Analysis

For histological staining, the free-floating slices were mounted onto polylysine-coated slides and stained with cresyl violet according to standard procedures [50].

For immunohistochemistry, the free-floating slices were treated for 25 min with 1% H_2_O_2_ diluted in Sorensen’s phosphate buffer (SPB, pH 7.35) for blocking endogenous peroxidase activity, and with blocking solution (SPB containing 0.3% Triton X-100, 2% normal horse serum) at room temperature (RT) for 60 min for blocking nonspecific binding sites. Subsequently, the slices were incubated with a rabbit anti-active caspase-3 antibody (1:50 in blocking solution) at 4 °C overnight, prior to washing for 2 × 5 min in SPB and incubating with a biotinylated secondary antibody (immunoglobulin IgG (H + L); 1:350 in blocking solution) for 2 h at room temperature. The streptavidin/biotin technique was employed for detection, using the ABC-Elite Kit with a 1 h incubation at 4 °C followed by a 1 h incubation at RT. For visualization, slices were incubated in 0.07% 3,3′-diaminobenzidine tetrahydrochloride/0.3% H_2_O_2_ in SPB (pH 7.2) until the brown staining was observed under the microscope. After washing, the slices were mounted onto polylysine-coated slides and dried overnight, and some slices were counterstained using hematoxylin solution acc. to Gill II.

The stained sections were dehydrated in a series of graded ethanol, processed through n-butylacetate, coverslipped with Entellan^®^ and left to dry overnight. Microscopic pictures were taken with a Zeiss Axioskop upright microscope (Carl Zeiss, Jena, Germany), equipped with a Zeiss AxioCam ICc 1 camera (Carl Zeiss, Jena, Germany).

### 2.13. In Vivo Distribution of the CaP-NPs by CED

#### 2.13.1. CaP-NP Preparation for CED

CaP-NPs were prepared with 3.75 mmol/L PO_4_^3−^ and loaded with 10 µmol/L AllStars Negative Control siRNA AF647. The oligomer was added according to method 2 with a concentration of 40 µmol/L. Due to the limitation of the injectable volume (5 µL), CaP-NPs were concentrated prior to CED injection using centrifugal ultrafiltration, as described above (Section 2.4). In total, 5 µL of concentrated NP dispersion was injected via CED in the brain of healthy animals.

#### 2.13.2. Convection-Enhanced Delivery

All experimental work including on animals was conducted in accordance with the national legislation on the use of animals for research (Tierschutzgesetz (TierSchG), Tierschutz-Versuchstierverordnung (TierSchVersV)) and was approved by the responsible research ethics committee (TVV 36/18 Landesdirektion Sachsen, 7 February 2019).

During the time of the experiment, animals were kept in a dedicated climatic chamber with free access to water and food under a 12:12 h dark/light cycle at a constant temperature of 24 °C.

Fischer rats, 8 weeks old (230–300 g), underwent volatile anesthesia with a mixture of air and isoflurane concentrate (1.5–2% depending on the breathing) during the microsurgery. The rats’ heads were fixed into a stereotactic frame (Motorized New Standard Stereotaxic^®^, Stoelting, Dublin, Ireland). A midline incision was created, and a burr hole was drilled 0.5 mm anterior and 3.6 mm lateral to the bregma. A volume of 5 µL AllStars Negative Control siRNA AF647 or concentrated CaP-NP dispersion was injected 6.0 mm into the brain parenchyma at a rate of 0.2 µL/min for 25 min using a customized 1-step cannula mounted on a 10 μL Hamilton syringe (30-gauge stainless needle protecting a fused-silica internal cannula (i.d. × o.d.: 75 µm × 150 µm) (Phymep, Paris, France) cut to expose only 2 mm length of the inner cannula). After injection, the burr hole was filled with bonewax (Ethicon, Bridgewater, NJ, USA), the scalp incision was sutured (Vicryl 6.0, Ethicon, Bridgewater, NJ, USA) and the surface was antiseptically cleaned.

#### 2.13.3. In Vivo Biodistribution

The animals were sacrificed 1 h after CED. Their brains were isolated and cryopreserved by incubation in 2-methylbutane at −25 °C. Cryo-sectioning into coronal sections of 12 µm thickness (Thermo Scientific Microm HM560, Schwerte, Germany) was performed, and sections were kept at −25 °C. Investigation of the CaP-NP biodistribution was assessed by fluorescence microscopy (Leica, DMi8, software Leica LASX, Leica Mikrosysteme Vertrieb GmbH, Wetzlar, Germany). The CaP-NP distribution was first assessed in the healthy parenchyma of rat brains. For the planned duration of 1 h, the first group received CaP-NPs loaded with 1 µmol/L of AllStars Negative Control siRNA AF647 (*n* = 2), and the second group received CaP-NPs loaded with 10 µmol/L of AllStars Negative Control siRNA AF647 (*n* = 2).

### 2.14. Statistical Analysis

Statistical analysis was performed by using GraphPad PRISM software (GraphPad Software Inc., La Jolla, CA, USA). Statistically significant differences between the different groups were analyzed by using a one-way or two-way ANOVA test with Tukey’s post hoc test. A *p*-value below 0.05 was considered statistically significant and is indicated with a single asterisk (*).

## 3. Results and Discussion

### 3.1. Synthesis and Characterization of the Terpolymer o14PEGMA(1:1:2.5)_NH_3_

**Figure 2:**
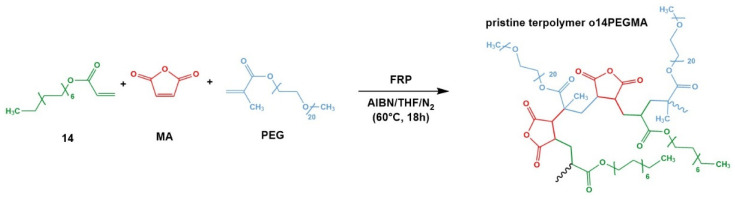
Synthesis of the amphiphilic terpolymer oligo(TDA-co-PEG-co-MA) (o14PEGMA) from tetradecylacrylate (14), methoxy poly(ethylene glycol) methacrylate (PEG) and maleic anhydride (MA) by free radical polymerization (FRP).

The amphiphilic oligomer was successfully synthesized according to the scheme in Figure 2.

The incorporation of the comonomer MA, which provides functional groups for chemical derivatization and the formation of anionic groups, was determined by conductometric and Brown–Fujimori titration with a mean value of 48.3 ± 5.8 mol% and a chemical intactness of 60.1% (Figure S1).

Proton NMR revealed the presence of all comonomers in the product (Figure S1) and was analyzed to obtain the amount of incorporated methoxy PEG methacrylate per molecule of TDA. In combination with the titration data for MA incorporation, the average comonomer composition of the synthesized o14PEGMA(1:1:2.5) was determined as 1:1.25:2.25. From our previous experience with MA-containing oligomers, we attribute the observed slightly overproportioned incorporation of PEG-MA and slightly underproportioned incorporation of MA to the reactivities of the different monomers. GPC revealed a number average molecular weight of 5600 ± 60 (Figure S2) and a dispersity of 2.0. On average, we expect an oligomer molecule to be composed of 3.34 TDA, 4.2 PEG-MA and 7.5 MA monomers per molecule.

### 3.2. Characterization of the CaP-NPs

**Figure 3:**
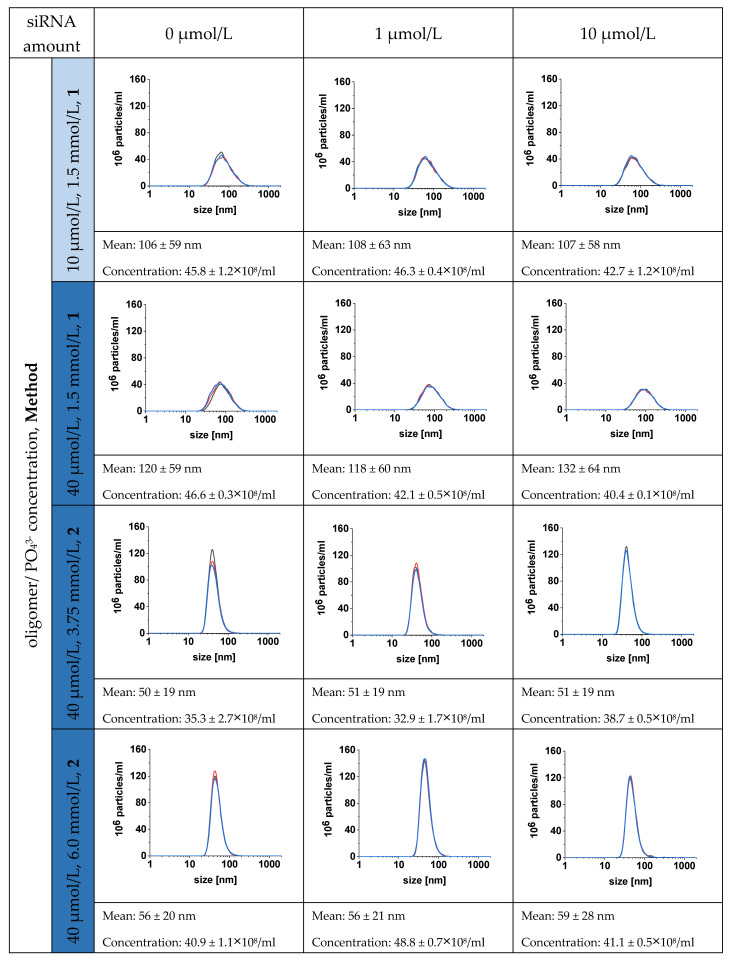
CaP-NP number distribution for different PO_4_^3−^ concentrations in the presence of 0, 1 and 10 µmol/L siRNA, and 10 and 40 µmol/L o14PEGMA(1:1:2.5)_NH_3_. For 1.5 mmol/L PO_4_^3−^, data are only presented for method 1 because only very few particles were formed with method 2, whereas for 3.75 and 6 mmol/L PO_4_^3−^, method 2 was chosen, which gave smaller CaP-NPs than method 1. Size measurements were performed by using NTA. *n* = 3.

Size and zeta potential are two crucial parameters influencing the in vivo distribution and uptake of NPs. In order to determine the influence of the terpolymer concentration on the size distribution of CaP-NPs, we started our investigations on o14PEGMA(1:1:2.5)_NH_3_ in a concentration range between 2 and 10 µmol/L (Figure S3). We chose laser diffractometry that enabled us to determine broad particle size distributions. This method allowed us to screen our formulations which may concomitantly contain NPs below 100 nm in size and aggregates or larger CaP particles up to the millimeter size range. Three different PO_4_^3−^ concentrations were used, i.e., 1.5, 3.75 and 6.0 mmol/L PO_4_^3−^, in agreement with other studies [20,21,22,23,26,32,51,52,53,54]. Independent of the used PO_4_^3−^ concentration, we found no stable formulations at oligomer concentrations of 2 and 5 µmol/L. The addition of 10 µmol/L oligomer showed NPs below 100 nm in size for 1.5 mmol/L PO_4_^3−^, but not for higher PO_4_^3−^ concentrations, where aggregates formed within 30 min (Figure S3). Obviously, a concentration of 10 µmol/L o14PEGMA(1:1:2.5)_NH_3_ was not able to fully cover the increased numbers of NPs precipitated with 3.75 or 6.0 mmol/L PO_4_^3−^ and protect them against aggregation. The need to increase the stabilizer concentration at higher PO_4_^3−^ concentrations has been reported before [55]. Consequently, we increased the oligomer concentration to 40 µmol/L (Figure 3 and Figure S4).

With the aim to collect additional information on the particle number, and due to the smaller sample volume required, we relied on NTA for further particle size measurements (Figure 3). This method provides microscopic scattering images to visualize aggregate formation, although no size information on the aggregates with a size beyond about 1000 nm can be provided. The increased stabilizer concentration (40 µmol/L) resulted in CaP-NP dispersions with an average size of about 50 nm for 3.75 and 6 mmol/L PO_4_^3−^ (Figure 3 and Figure S4). Similar correlations between the stabilizer concentration and CaP-NP size have been reported before for other stabilizers [21,22]. Giger et al. (2013) employed an alendronate-derivatized PEG2000 (PEG-Ale) as a stabilizer for CaP-NPs and compared it to inositol pentakisphosphate-derivatized PEG2000 (PEG-IP5) [21]. Preparation of CaP-NPs with a PEG-Ale concentration of 5 µmol/L for 0.5 mmol/L PO_4_^3−^ resulted in CaP-NPs of about 190 nm in size that rapidly grew to more than 500 nm after three days. As they increased the PEG-Ale concentration to 10 µmol/L for 0.5 mmol/L PO_4_^3−^, the authors obtained stable CaP-NP formulations with an average size of 260 nm after one month of storage.

Huang et al. (2017) reported similar findings using PEG-IP5 with a PEG length of 5 kDa. In that study, they screened for the impact of different PEG-IP5 concentrations (5, 10, 20, 30, 40 µmol/L) on CaP-NP size prepared with different PO_4_^3−^ concentrations (1.5, 3, 4, 5, 6 mmol/L) [22]. For each of the applied PO_4_^3−^ concentrations, they found a steady decrease in the average size with an increase in the PEG-IP5 concentration. As observed in our study, the addition of 10 µmol/L polymer to CaP-NPs prepared from 1.5 mmol/L PO_4_^3−^ was sufficient to obtain NPs smaller than 200 nm.

Beside the effect of the oligomer concentration, we also observed that the preparation method impacted CaP-NPs’ stability. Two different methods were investigated: method 1, where CaP-NP precipitation was immediately followed by the addition of the stabilizer, and method 2 that involved precipitation in the presence of the stabilizer (Figure S4). With PO_4_^3−^ concentrations higher than 1.5 mmol/L, we found that only method 2 resulted in NPs smaller than 100 nm. Since, for method 1, the stabilizer was added after mixing of Ca^2+^ and PO_4_^3−^ solutions, we assumed that so-called prenucleation clusters between Ca^2+^ and PO_4_^3−^ were formed, building up loosely aggregated networks [22]. By incorporating more ions from the solution, they would grow into densely aggregated spheres until a stabilizer is added [22]. With the PO_4_^3−^ concentration increased to 3.75 or 6.0 mmol/L, more prenucleation clusters were formed, which grew more rapidly due to the higher ion concentration. The addition of the oligomer after 4 s (method 1) therefore resulted in larger particles than for 1.5 mmol/L PO_4_^3−^. Furthermore, we observed that method 2 with 1.5 mmol/L PO_4_^3−^ caused a clear reduction in particle concentrations in comparison to method 1 (Figure S4). Considering these results, we speculate that a competition between the anionic oligomer and the phosphate ions for Ca^2+^ suppressed the formation of CaP prenucleation clusters and caused the growth of the few CaP particles via Ca^2+^ release from the oligomer, probably due to the higher thermodynamic stability of the CaP particles. In contrast, when method 1 was applied at a low PO_4_^3−^ concentration, CaP-NPs precipitated, and the oligomer added a few seconds after precipitation could bind to the CaP-NP surface and stabilize it.

In contrast to other studies, our oligomer contained a low-molecular weight PEG of 950 Da. This is known to reduce the steric stabilization effect and hence the colloidal stability of our oligomer-stabilized CaP-NPs [21]. Increasing the PEG chain length would lead to more stable CaP-NPs but would also cause a decreased cellular uptake [21,22,51,54]. To circumvent these storage stability issues, we developed a simple on-demand mixing reaction for the preparation of o14PEGMA(1:1:2.5)_NH_3_-stabilized CaP-NPs that allows for immediate application. We considered formulations as stable when the particle size remained unchanged 5 h after preparation.

**Figure 4:**
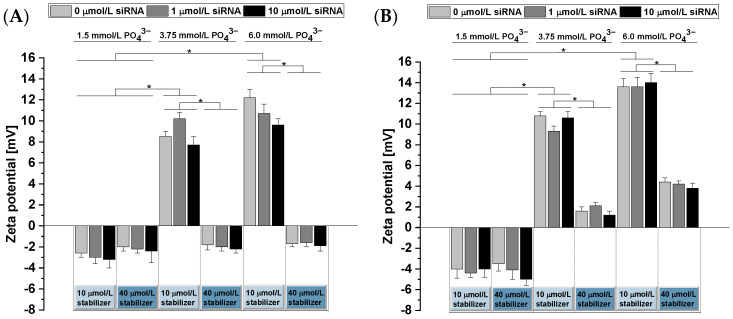
Zeta potential measurements of o14PEGMA(1:1:2.5)_NH_3_-stabilized CaP-NPs prepared via methods 1 (**A**) and 2 (**B**). PO_4_^3−^ and oligomer concentrations and siRNA amounts were systematically varied. Data are presented as mean ± SD (*n* = 3), * *p* < 0.05.

In addition to the analysis of the particle size, we performed zeta potential measurements (Figure 4). For CaP-NPs with 1.5 mmol/L PO_4_^3−^, the zeta potential was found to be in the weakly negative range independent of the siRNA concentration as well as the chosen precipitation method when oligomer concentrations of at least 10 µmol/L were added. In contrast, for higher PO_4_^3−^ concentrations (3.75 and 6.0 mmol/L) that concomitantly resulted in higher numbers of NPs, we observed a positive zeta potential with 10 µmol/L oligomer and increasing positive zeta potentials with increasing PO_4_^3−^ concentrations, suggesting an insufficient surface coverage with the stabilizer. An increase in the oligomer concentration to 40 µmol/L, however, restored the neutral zeta potential found with higher oligomer-to-PO_4_^3−^ concentrations. Methods 1 and 2 caused slightly negative and slightly positive zeta potentials, respectively, that were significantly higher for 6 mmol/L than for 3.75 mmol/L PO_4_^3−^. This strongly indicates an adsorption of the oligomer to the CaP-NPs. The slight differences between methods 1 and 2 may indicate greater surface formation with the use of method 2 because crystallization nuclei can be stabilized immediately upon formation, which eventually requires a greater amount of the oligomer to fully stabilize the NP surface. The addition of siRNA had no measurable effect on the zeta potential, which may be explained via the dominant PEG effects after adsorption of the oligomer that covered the underlying siRNA effects, but it may also indicate that siRNA was not bound. To ensure that siRNA remains incorporated in the o14PEGMA(1:1:2.5)_NH_3_-stabilized CaP-NPs, we analyzed the siRNA binding capacity of our stabilized CaP-NPs in the next step.

### 3.3. Determination of the siRNA Binding Capacity

**Figure 5:**
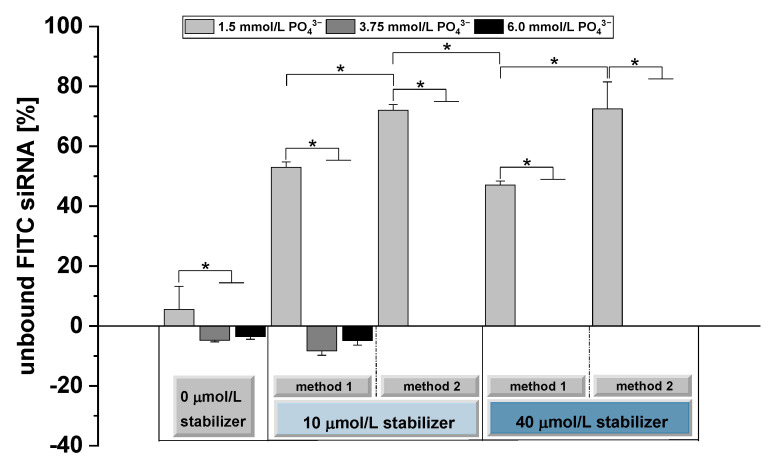
Analysis of FITC-siRNA binding capacity of o14PEGMA(1:1:2.5)_NH_3_. Different formulations of CaP-NPs were prepared and loaded with 1 µmol/L FITC-siRNA. The amount of unbound FITC-siRNA was determined after centrifugal filtration and normalized to the control (1 µmol/L FITC-siRNA without CaP-NPs). Data are presented as mean ± SD (*n* = 4), * *p* < 0.05.

Since we found no significant influence of siRNA loading on the size distribution and zeta potential (Figure 3 and Figure 4), we determined the siRNA binding capacities of different CaP particle formulations in the absence and presence of the oligomer. For analysis, the particles were loaded with FITC-labeled siRNA. Unbound FITC-siRNA was separated from particle-bound FITC-siRNA by ultrafiltration (Amicon Ultra-4 100K, Merck Millipore, Tullagreen, Ireland). The amount of unbound FITC-siRNA for the different formulations is shown in Figure 5.

In the absence of o14PEGMA(1:1:2.5)_NH_3_, CaP-NPs showed high FITC-siRNA binding capacities independent of the respective PO_4_^3−^ concentration (1.5/3.75/6.0 mmol/L). For 3.75 and 6.0 mmol/L PO_4_^3−^, the addition of 10 or 40 µmol/L stabilizer had no impact on the FITC-siRNA binding. Independent of the preparation method, we could not detect any unbound FITC-siRNA after ultrafiltration (Figure 5).

In contrast, we found high amounts of unbound FITC-siRNA for 1.5 mmol/L PO_4_^3−^ after the addition of 10 or 40 µmol/L stabilizer (Figure 5), whereas no unbound siRNA was determined in the absence of the stabilizer for 1.5 mmol/L PO_4_^3−^. We therefore assumed that for the limited CaP-NP surface provided by 1.5 mmol/L PO_4_^3−^, the oligomer addition interferes with siRNA binding to CaP. Kakizawa et al. (2004) reported similar observations by applying PEG-block-poly(aspartic acid) (PEG-PAA) for inhibition of CaP crystal growth [23]. Preparation of CaP-NPs with 3.0 mmol/L PO_4_^3−^ showed 97% oligodeoxynucleotide (ODN) binding, whereas for 1.5 mmol/L PO_4_^3−^, the amount of incorporated ODN substantially decreased with increasing PEG-PAA concentrations [23]. In contrast, Giger et al. (2013) found no siRNA displacement for PEG-alendronate (5 or 10 µmol/L) and PEG-inositolpentakisphosphate (10 µmol/L) at 1.5 mmol/L Na_2_HPO_4_ [21].

These reported divergent effects indicate that larger anionic blocks may bind more efficiently to the CaP-NPs than small, high-affinity block copolymers with PEG, where the PEG-block may interfere with the dense packaging of the stabilizer on the NPs.

Beside the competition between the stabilizer and siRNA, the CaP ratio seems to be another crucial factor influencing the siRNA binding efficiency in our study. Applying 1.5 mmol/L PO_4_^3−^ for particle preparation resulted in clearly lower particle concentrations than for the lower Ca/P ratios using 3.75 and 6.0 mmol/L PO_4_^3−^ (Figure 3 and Figure S4).

The effect of the particle concentration on siRNA binding was further illustrated by comparing both preparation methods at 1.5 mmol/L PO_4_^3−^. Whereas preparation method 1 showed an siRNA binding efficiency of about 50%, the binding efficiency during preparation method 2 significantly decreased to about 30% (Figure 5). Since, with method 1, we always found less free siRNA than with method 2, we assumed that siRNA, once bound, is less easily displaced by the oligomer than with method 2, where competition takes place from the initiation of particle formation. Studying the impact of the Ca/P ratio on the binding of siRNA-mimicking Cy3-dsDNA to lipid-coated CaP-NPs, Tang et al. (2015) found the highest encapsulation efficiency at a low Ca/P ratio that decreased with an increasing CaP ratio probably caused by the amounts of particles formed [55].

Taken together, o14PEGMA(1:1:2.5)_NH_3_-stabilized CaP-NPs showed high and efficient binding of FITC-siRNA depending on the used Ca/P ratio, making formulations with more than 1.5 mmol/L PO_4_^3−^ suitable as an siRNA delivery system.

### 3.4. Determination of the Oligomer Binding Capacity

As a next step, we determined the amount of o14PEGMA(1:1:2.5)_NH_3_ bound to siRNA-loaded CaP-NPs. To this end, we labeled the oligomer with different amounts of Cy5-amine (5, 10, 20, 40 µmol/L) via a partial aminolysis of maleic anhydrides before the remaining anhydride groups were hydrolyzed to generate additional carboxylic acid groups required for binding to CaP.

Since the labeling of the oligomer with Cy5-amine via maleic anhydrides could decrease the affinity to CaP, we analyzed the stability of CaP-NPs prior to the binding capacity study. Fluorescence microscopy was used to investigate the aggregate formation due to the possibly impaired stabilization efficiency (data not shown). Only with the lowest amount (5 µmol/L) of Cy5-amine did we find no aggregate formation and unchanged zeta potential values (data not shown), whereas higher substitution grades resulted in an increased tendency for aggregate formation probably caused by a decrease in free carboxylic acid groups required for proper NP stabilization. Moreover, these results clearly show the importance of hydrolyzed maleic anhydrides for CaP-NP stabilization.

In a similar approach to that used for siRNA binding analysis, we investigated the binding of o14PEGMA(1:1:2.5)_NH_3_ labeled with 5 µmol/L Cy5-amine to CaP-NPs. For the separation of the unbound (flow-through) stabilizer from CaP-NPs (retentate), we applied ultrafiltration using centrifugal filter units (Amicon Ultra-4 100K, Merck Millipore, Tullagreen, Ireland).

**Figure 6:**
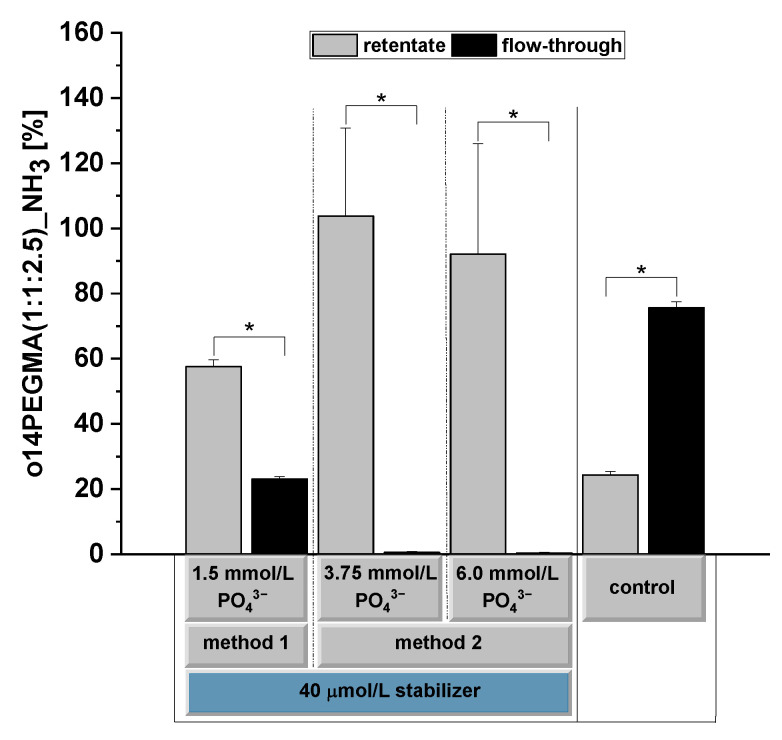
Analysis of teroligomer binding to siRNA-loaded CaP-NPs via o14PEGMA(1:1:2.5)_NH_3_ _Cy5-amine. CaP-NPs were prepared with 1.5, 3.75 or 6.0 mmol/L PO_4_^3−^ and stabilized with 40 µmol/L o14PEGMA(1:1:2.5)_NH_3__Cy5-amine. CaP-NPs were loaded with 1 µmol/L siRNA. After centrifugal ultrafiltration, the fluorescence intensities of the retentates and the flow-throughs were determined and normalized to the total fluorescence intensity of each group. As a control, the dissolved oligomer was filtered in the absence of CaP-NPs: The dissolved Cy5-amine-labeled stabilizer could fully pass through the centrifugal filter units. To also measure the retentate for comparison with the CaP-NP groups, we retained a volume fraction on the centrifugal filter units. Data are presented as mean ± SD (*n* = 4), * *p* < 0.05.

We investigated the binding of the oligomer to CaP-NPs with different NP concentrations resulting from different PO_4_^3−^ concentrations. As a positive control for the unbound teroligomer, we used a cyanine-5-amine-labeled stabilizer at the same concentration as that used for CaP-NP stabilization. We integrated this control in our experiment to exclude any binding of the oligomer to the centrifugal filter units. Figure 6 shows that the oligomer easily passed through the filter. We kept a small volume of the solution to generate a retentate as a control. The retentate and flow-through summed up to 100% of the oligomer. For 3.75 and 6 mmol/L PO_4_^3−^, we detected no fluorescence in the flow-through, indicating that the complete amount of the oligomer was tightly bound to the NPs. For 1.5 mmol/L PO_4_^3−^, however, about 25% of the applied oligomer remained free, probably after the NP surface was covered with the oligomer caused by the reduced particle numbers. This is in agreement with the zeta potential results reported above.

The results for the siRNA (Figure 5) and oligomer binding (Figure 6) confirm the high affinity binding of both and support the suitability of the oligomer-stabilized CaP-NPs as carriers for siRNA, with a dependency on the used PO_4_^3−^ concentration. If the ratio between the oligomer and precipitated NPs increases critically, as shown for 1.5 mmol/L PO_4_^3−^, especially with method 2, reduced amounts of siRNA bind to the surface, indicating that the oligomer competes with siRNA for the CaP-NP surface.

To our knowledge, this is the first time that the actual amount of the stabilizer binding to CaP-NPs has been reported. Although several studies showed an impact of stabilizer addition on the surface charge [31,32,56], the proper amount of added stabilizer that binds to CaP-NPs remained unclear, and any impact of the free stabilizer on the zeta potential cannot be excluded in these studies.

### 3.5. Determination of the Serum Stability

To assess the stability of our siRNA-loaded CaP-NPs in the transfection medium (DMEM high glucose + 2% FBS), we monitored the particle size continuously during the transfection procedure (5 h). NTA measurements revealed no significant impact of the cell culture medium and serum proteins on the particle sizes of the investigated formulations (Figure S5).

These findings are in agreement with the results of other studies that demonstrated the stability of stabilized CaP-NP systems in the presence of serum proteins [21,22,26,28,30,51,56]. To analyze the silencing efficiency and functional stability of oligomer-stabilized CaP-NPs, we determined in vitro silencing efficiencies in the next step.

### 3.6. Determination of In Vitro Silencing

We performed in vitro transfection experiments to determine the siRNA silencing efficiency in the rat glioblastoma cell line F98. As a first molecular target, we chose BIRC5, which encodes the inhibitor of the apoptosis protein survivin, and determined the BIRC5 mRNA expression levels on day 2 post-transfection as well as cell viabilities on day 3 post-transfection.

**Figure 7:**
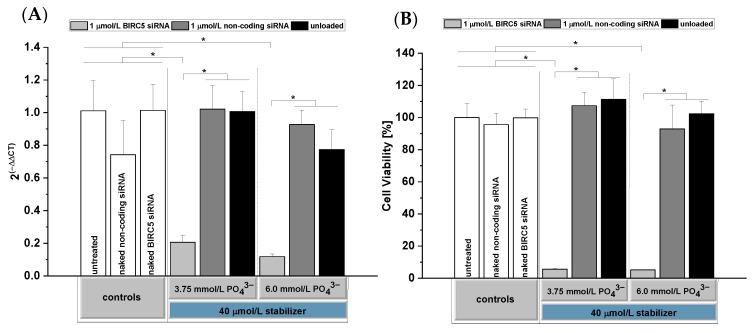
In vitro siRNA silencing efficiency in F98 cells of o14PEGMA(1:1:2.5)_NH_3_-stabilized siRNA-loaded CaP-NPs (**A**) on day 2 post-transfection determined by using quantitative real-time PCR, and (**B**) cell viability of F98 cells on day 3 post-transfection determined via the WST-8 assay. Particles were prepared with 3.75 mmol/L PO_4_^3−^ or 6.0 mmol/L PO_4_^3−^ and 40 µmol/L o14PEGMA(1:1:2.5)_NH_3_ according to method 2. Particles were loaded with 1 µmol/L BIRC5 siRNA. Data were normalized to untreated cells and are presented as mean ± SD (*n* = 4), * *p* < 0.05.

As shown in Figure 7, formulations with 3.75 and 6 mmol/L PO_4_^3−^ resulted in high silencing efficiencies of BIRC5, whereas no significant silencing was observed in the control groups including CaP-NPs with non-coding siRNA as well as naked siRNA. No significant influence of the Ca/P ratio on the silencing efficiency was observed for 3.75 and 6 mmol/L PO_4_^3−^. The strong decrease in BIRC5 mRNA expression finally resulted in a strong decrease in cell viability (to ~5%) on day 3 post-transfection (Figure 7B). A positive control with Lipofectamine^TM^ RNAiMAX had shown a slightly lower silencing efficiency in other experiments (data not shown). However, with Lipofectamine^TM^, clearly less siRNA is recommended for silencing by the manufacturer. We therefore considered this approach as not suitable as a positive control and dispensed with this group in the shown experiment.

This remarkable silencing effect relies on a combination of efficient cellular uptake supported by our stabilizer and a well-documented endosomal release mechanism: Due to the acid environment, CaP-NPs are rapidly dissolved in late endosomes, leading to an increased concentration of calcium and phosphate ions. This increased osmotic pressure leads to water influx, causing rupture of the endosomal membrane and release of siRNA into the cytosol [21,57,58].

Concerning the uptake efficiency, other studies described reduced cellular uptakes by increasing the concentration of PEGylated stabilizers with a larger PEG molecular weight [20,21,22,54].

Distinct from most other studies using PEG-containing stabilizers, we applied a shorter PEG length of 950 Da. In consequence, cellular uptake remained almost unimpaired after stabilization with o14PEG-MA up to concentrations of 40 µmol/L for 3.75 and 6 mmol/L PO_4_^3−^. However, this shorter PEG length clearly reduced the colloidal stability of our NPs compared to other studies involving stabilizers with longer PEG chains. Nonetheless, we ensured the stability of particle sizes over 5 h that should be sufficient to prepare the CaP-NPs and administer them via CED.

Since CaP-NPs prepared with 3.75 or 6.0 mmol/L PO_4_^3−^ showed no differences in siRNA silencing efficiency, we used 3.75 mmol/L PO_4_^3−^ for analysis of apoptosis induction, biocompatibility testing and preliminary in vivo distribution studies.

### 3.7. Effects of BIRC5 Silencing on Apoptosis Induction

**Figure 8:**
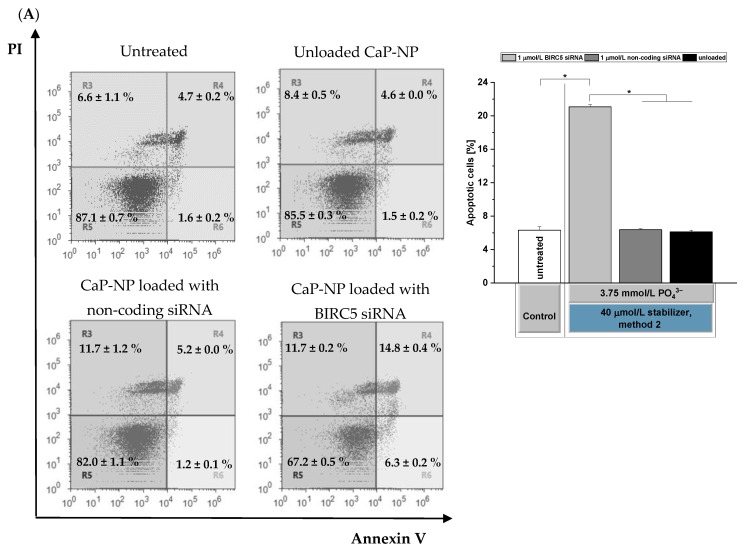

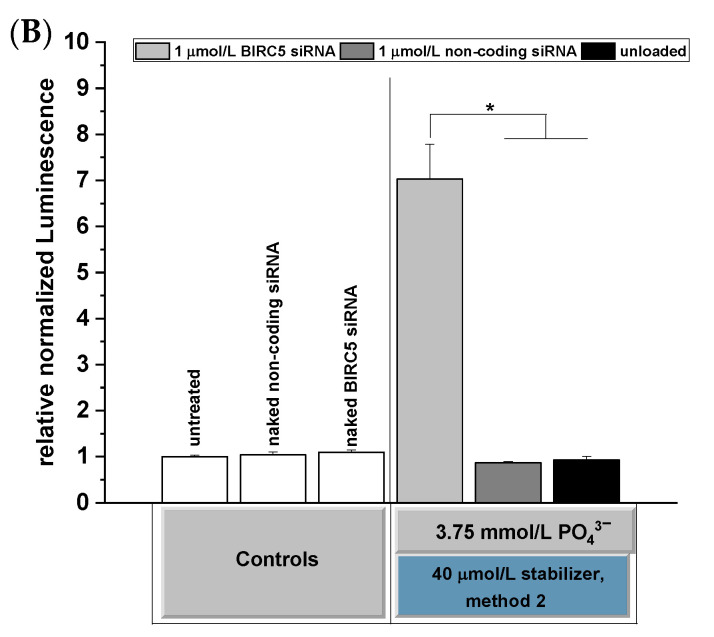
Analysis of BIRC5 silencing-mediated apoptosis induction in F98 cells via BIRC5 siRNA-loaded CaP-NPs (**A**) on day 2 post-transfection determined by using Annexin V/propidium iodide staining and (**B**) Caspase-Glo^®^ 3/7 assay on day 3 post-transfection. The figure shows luminescence per WST metabolic activity. Data were normalized to the untreated control. Particles were prepared with 3.75 mmol/L PO_4_^3^^−^ and 40 µmol/L o14PEGMA(1:1:2.5)_NH_3_ according to method 2. Data are presented as mean ± SD (*n* = 4), * *p* < 0.05.

To investigate if the decreased cell viability (Figure 7B) upon successful BIRC5 silencing (Figure 7A) is caused by apoptosis induction in F98 cells, we employed a caspase assay and normalized the results to the metabolic activity since we observed reduced cell numbers after BIRC5 silencing. We found strongly induced caspase-3/7 activity (seven times higher) for BIRC5 siRNA-loaded CaP-NPs after normalization to the metabolic activity relative to all negative controls (Figure 8B). In addition, we applied Annexin V and propidium iodide staining on day 2 post-transfection. Flow cytometric analysis showed that 21% of the cells were apoptotic (Annexin V+/PI− or positive for both; sum of squares R4 and R6). In controls, we found that 6% of the cells were apoptotic (Figure 8A). The high proportion of Annexin V+/PI+ cells indicated that F98 cells were at the late stage of apoptosis caused by the loss of membrane integrity [59,60,61]. These results clearly show an induction of apoptosis in F98 cells caused by BIRC5 silencing mediated by the oligomer-stabilized CaP-NPs.

### 3.8. In Vitro Cytocompatibility Testing

In vitro cytocompatibility testing was performed according to ISO 10993-5 [40] using L929 mouse fibroblasts. L929 cells were incubated with stabilized CaP-NPs for 24 h.

We tested CaP-NPs with 1 µg siRNA/well in 75 µL of HEPES buffer as well as 5 µL of ultrafiltration-concentrated CaP-NPs with 10 µg siRNA/well, which was used in the in vivo study, too. After 24 h, CaP-NPs were removed, and cells were kept for another 48 h to analyze the recovery potential. The analysis was performed by using Rotitest^®^ Vital (WST-8 assay) based on formazan formation by viable cells.

After 24 h of incubation, the metabolic activity of treated L929 cells was higher than 70% for CaP-NPs, whereas no significant effect was found 72 h after the initial exposure.

**Figure 9:**
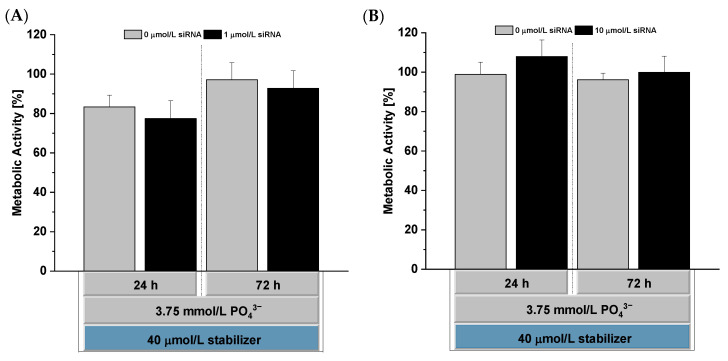
In vitro cytocompatibility of o14PEGMA(1:1:2.5)_NH_3_-stabilized siRNA-loaded CaP-NPs ((**A**): non-concentrated, (**B**): ultrafiltration-concentrated) immediately after the initial exposure (24 h value) and after 48 h of recovery in fresh culture medium (72 h value) determined via the WST-8 assay. Data were normalized to the metabolic activity of untreated L929 cells (100% activity) and are presented as mean ± SD (*n* = 4).

Using concentrated CaP-NPs, we found no significant influence on the metabolic activity for both investigated time points (Figure 9). Live/Dead staining further showed no significant influence on the cell viability of L929 cells (Figure S7). Further investigations need to show if neuronal cells would also tolerate our NPs. For a first insight into tissue-specific effects, we investigated cytotoxicity in organotypic brain slice cultures in the next step.

### 3.9. Absence of Adverse Effects of CaP-NPs in Organotypic Brain Slice Cultures

Beyond 2D tissue culture, the analysis of possible effects of nanoparticles on intact tissue integrity and cell viability is relevant as well. This is particularly true for neuronal tissue.

To assess the possible toxicity of CaP-NPs on neuronal tissue, we employed tissue slices prepared from mouse brains that were cultivated in an air–liquid interface culture.

**Figure 10:**
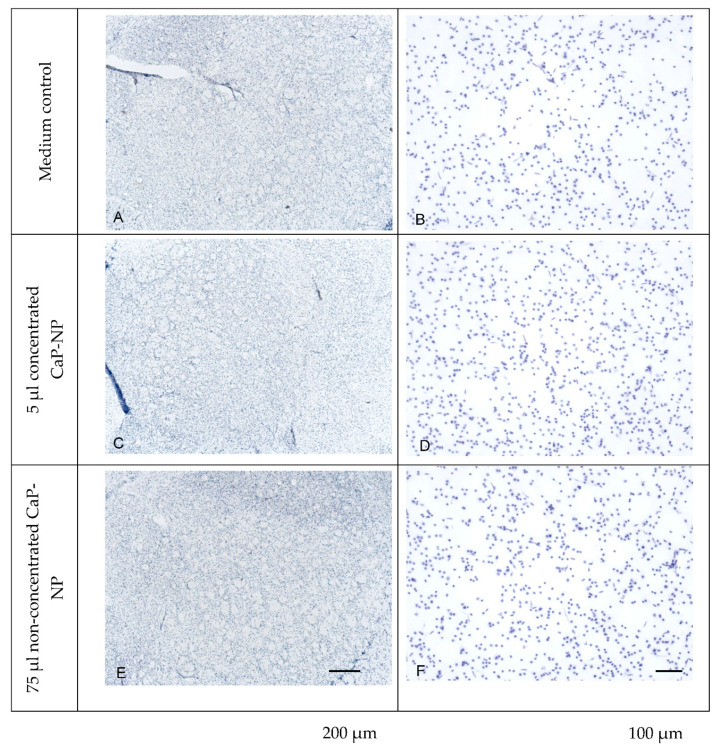
Histological analysis of tissue slices from neuronal tissue, cultivated in an air–liquid interface culture. Cresyl violet nuclei staining of sections from tissue samples in the absence (**A**,**B**) or presence of non-concentrated (**C**,**D**) or concentrated (**E**,**F**) CaP-NPs is shown at two magnifications (scale bars: (**A**,**C**,**E**) = 200 µm; (**B**,**D**,**F**) = 100 µm).

After 72 h of cultivation in the presence or absence of nanoparticles, the tissue integrity of the slices was analyzed by cresyl violet staining. This staining of nuclei revealed no difference in the density or distribution pattern of neuronal cells in the mouse striatum, indicating the preservation of tissue integrity upon incubation with low or high concentrations of nanoparticles as compared to the negative control (i.e., the same medium without nanoparticles; Figure 10).

**Figure 11:**
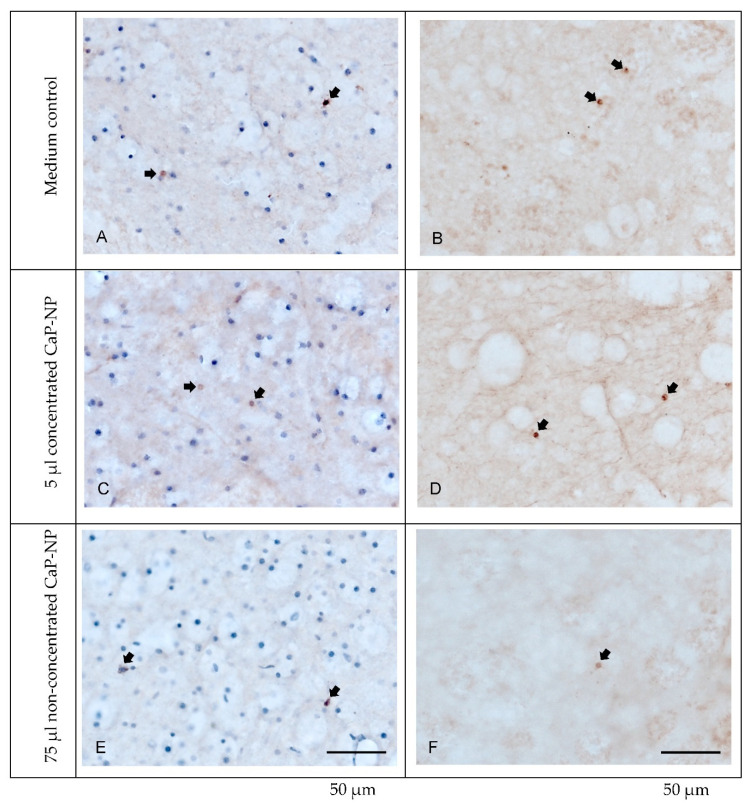
Immunohistochemical analysis of active caspase-3 as an indicator of apoptotic cell death. Sections from tissue samples cultivated in the absence (**A**,**B**) or presence of non-concentrated (**C**,**D**) or concentrated (**E**,**F**) CaP-NPs, with (**left**) or without hematoxylin counterstain (**right**). Positive nuclei (brown) are indicated by the arrows (scale bars: 50 µm).

This notion was also confirmed by immunohistochemical analysis of cleaved caspase-3 as a pivotal mediator of both intrinsic and extrinsic apoptosis (Figure 11). The negative control revealed a very low number of apoptotic cells, as indicated by brown nuclei (see arrows in Figure 11). Notably, identical staining patterns were observed in the treated slices, with no increase in apoptotic cell numbers. Additionally, counterstaining with hematoxylin again revealed no alterations in the overall number and distribution of nuclei (Figure 11, left panel).

In line with this, the quantitation of LDH in the incubation medium from every well indicated no increase in LDH release upon nanoparticle treatment (data not shown), thus supporting the absence of acute toxicity.

### 3.10. Preliminary In Vivo Distribution Study

We performed a first in vivo experiment to study the distribution of CaP-NPs in healthy rat brain tissue via CED 1 h after administration (Figure S8). Suitable volumes and infusion rates had been experimentally determined before with a dye test solution to minimize backflow events (data not shown). For the NP distribution experiment, we increased the siRNA loading concentration to 10 µmol/L AllStars Negative Control siRNA AF647. This concentration had been determined to result in a higher fluorescence intensity in cellular uptake studies (Figure S6) compared to the use of 1 µmol/L. In histological brain sections, we determined fluorescence spots close to the injection site but no homogenous distribution in the brain tissue. We hypothesized that these spots may indicate cellular uptake of CaP-NPs (Figure S8) as they resemble the cell staining observed in Figure S6 during the in vitro cellular uptake study. In contrast, when free siRNA was applied in the absence of CaP-NPs, we found a homogeneous staining in the brain sections. Due to these results, we hypothesized that the missing tissue staining by CaP-NP-bound siRNA can be explained by a quenching effect that revealed the siRNA only after release from the CaP-NPs in late endosomes or after endosomal escape.

## 4. Conclusions

This study shows that the newly developed terpolymer o14PEGMA(1:1:2.5)_NH_3_ is able to stabilize CaP-NPs with a size of less than 100 nm and an almost neutral zeta potential. A simple method for the preparation of siRNA-loaded CaP-NPs for the immediate application of siRNA-carrying CaP-NPs was established. Stabilized by the terpolymer, siRNA-loaded CaP-NPs were able to efficiently silence survivin in F98 rat brain cancer cells. With low PO_4_^3−^ concentrations, resulting in low precipitation and low numbers of CaP-NPs, we observed competition between negatively charged siRNA and the terpolymer for the CaP-NP surface and, consequently, loss of silencing efficiency. The investigation of the cytotoxicity showed only minor and transient effects on L929 cells. No adverse effects were observed in ex vivo organotypic brain slice cultures. However, additional in vitro as well as in vivo studies need to be performed to elucidate the effects of CaP-NPs on neuronal cells, too.

## Figures and Tables

**Table 1:** Overview of terpolymer concentrations used for stabilization of CaP-NPs.

	Stock Solution (Used for CaP-NP Preparation)	Final Concentration (in Formulation)
**o14PEGMA(1:1:2.5)_NH_3_**	2 µmol/L	0.7 µmol/L
	5 µmol/L	1.7 µmol/L
	10 µmol/L	3.3 µmol/L
	40 µmol/L	13.3 µmol/L

**Table 2:** Overview of siRNA amounts used for loading of oligomer-stabilized CaP-NPs and siRNA transfection.

		Stock Solution (Used for Loading of CaP-NPs)	Final Amount/Well
**non-concentrated** **(48-well)**	BIRC5 siRNA	20 µmol/L	1.0 µg
	AllStars Negative Control siRNA	20 µmol/L	1.0 µg
	AllStars Negative Control siRNA AF647	20 µmol/L	1.0 µg
		200 µmol/L	10.0 µg
**Concentrated** **(96-well)**	BIRC5 siRNA	200 µmol/L	10.0 µg
	AllStars Negative Control siRNA	200 µmol/L	10.0 µg
	AllStars Negative Control siRNA AF647	200 µmol/L	10.0 µg

## Data Availability

Data available on request.

## Supplementary Materials

The following are available online at https://www.mdpi.com/article/10.3390/pharmaceutics14020326/s1, Figure S1: ^1^H-NMR of o14PEGMA (1:1:2.5) in CDCl_3_ with 0.03%TMS. Figure S2: Gel permeation chromatography chromatograms of o14PEGMA(1:1:2.5) to determine size distribution. Figure S3: Influence of oligomer concentration on size distribution and aggregation tendency of CaP particles determined by advanced Laser diffraction analysis. Figure S4: NTA screenshots of prepared CaP-particles. Figure S5: Effect of serum proteins and cell culture medium on stability of o14PEGMA(1:1:2.5)_NH_3_-stabilized CaP-NP after 5 h. Figure S6: Uptake of o14PEGMA(1:1:2.5)_NH_3_-stabilized CaP-NP in F98 cells. Figure S7: In vitro biocompatibility testing of CaP-NP in L929 cells: Live/Dead staining. Figure S8: In vivo biodistribution of CaP-NP loaded with 10 μmol/L AllStars Negative Control siRNA AF647.
