# Supplementary material for: Amphiphilic Anionic Oligomer-Stabilized Calcium Phosphate Nanoparticles with Prospects in siRNA Delivery via Convection-Enhanced Delivery
Source: Pharmaceutics. 2022 Jan 29;14(2):326. doi: 10.3390/pharmaceutics14020326 (PMC8877163; doi:10.3390/pharmaceutics14020326)
Supplement: Supplementary file 1 [file pharmaceutics-14-00326-s001.zip › pharmaceutics-1537777-supplementary.pdf]

pharmaceutics

Supplementary Materials: Amphiphilic Anionic Oligomer-Stabilized Calcium Phosphate Nanoparticles with Prospects in siRNA Delivery via Convection-Enhanced Delivery

Franziska Mitrach, Maximilian Schmid, Magali Toussaint, Sladjana Dukic-Stefanovic, Winnie Deuther-Conrad, Heike Franke, Alexander Ewe, Achim Aigner, Christian Wölk, Peter Brust, Michael C. Hacker and Michaela Schulz-Siegmund

Structural analysis of pristine oligomer

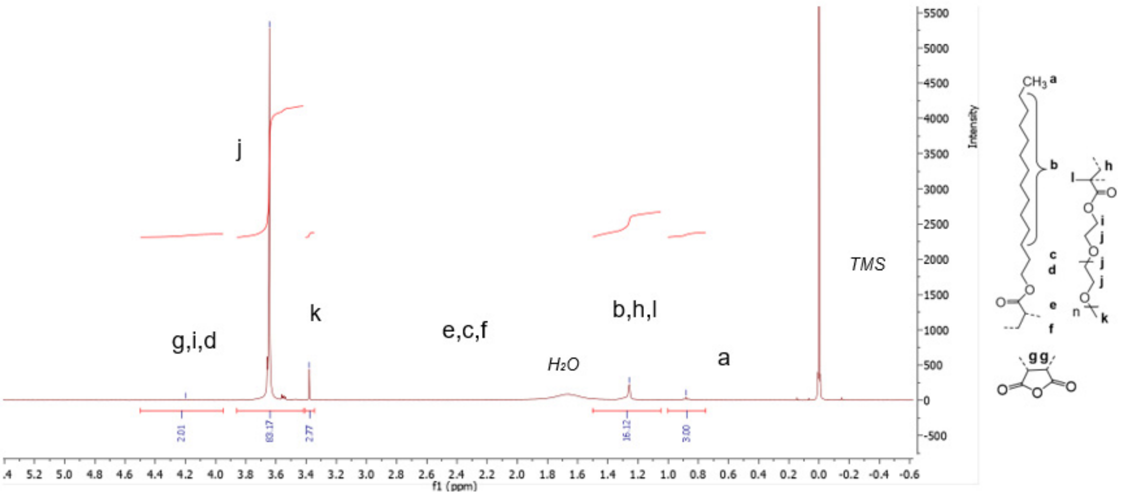

Figure S1. ^{1}H-NMR of o14PEGMA (04/04/10) in $CDCl_3$ with 0,03%TMS, c = 0.5 mg/ml

δ [ppm] 0.75-1.00 ;1.05-1.50; 3.35-3.40; 3.42-3.86; 3.95-4.50

Size exclusion chromatography of pristine oligomer stabilizer

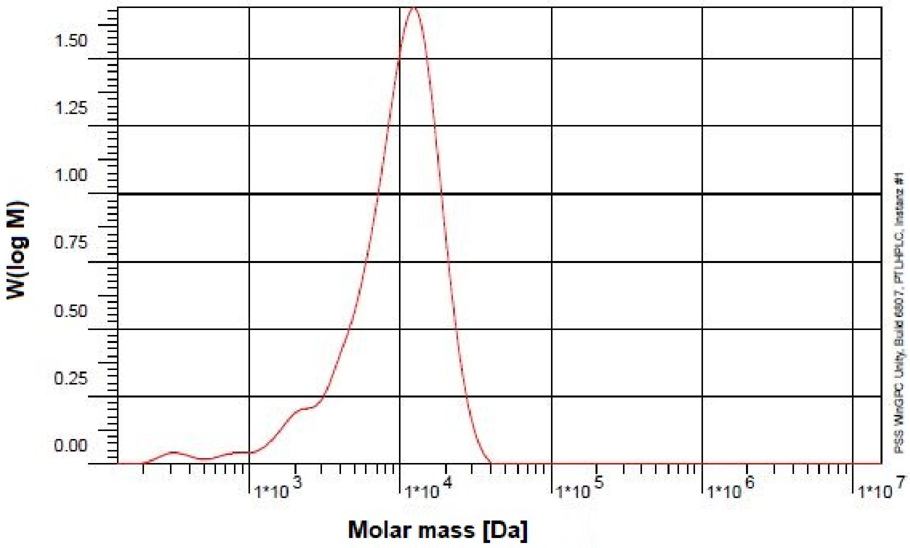

Figure S2. Gel permeation chromatography chromatograms of o14PEGMA(1:1:2.5) to determine size distribution c = 10mg/ml in THF ; V = 40 µl; flow rate=1ml/min; n=3

Impact of oligomer concentration on CaP-NP stabilization.

			Size distribution	
			**t = 5 min**	**t = 30 min**
**2 µmol/L o14PEGMA(1:1:2.5)_NH_3**	**1.5 mmol/L PO_4^{3-}**	**Method 1**	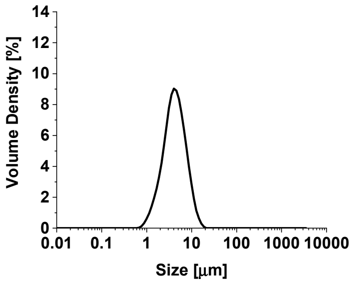<br>D[4;3] = 5.05 µm	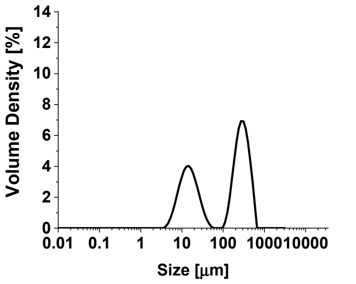<br>D[4;3] = 194 µm
		**Method 2**	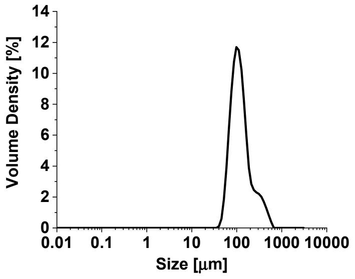<br>D[4;3] = 151 µm	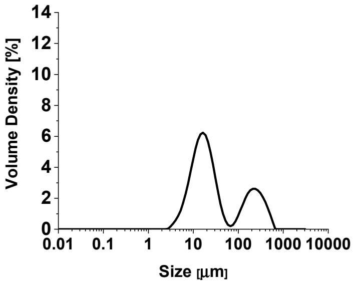<br>D[4;3] = 88.4 µm
	**6.0 mmol/L PO_4^{3-}**	**Method 1**	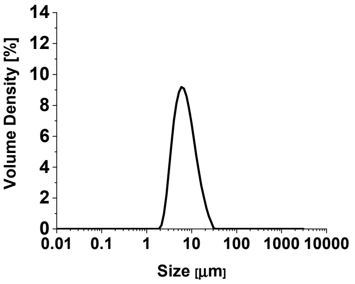<br>D[4;3] = 8.45 µm	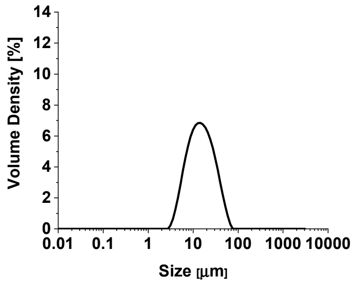<br>D[4;3] = 19.1 µm
		**Method 2**	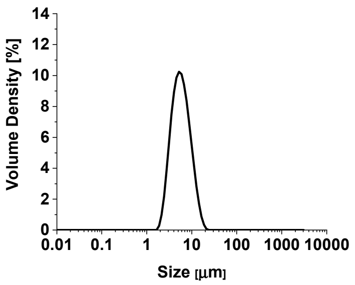<br>D[4;3] = 6.72 µm	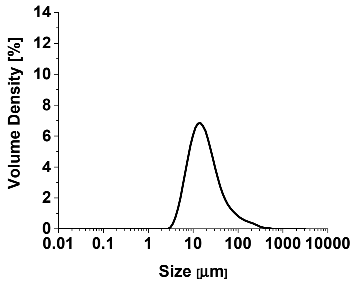<br>D[4;3] = 29.8 µm

<table>
<tr><th colspan="3"></th><th colspan="2">Size distribution</th></tr>
<tr><th colspan="3"></th><th>t = 5 min</th><th>t = 30 min</th></tr>
<tr><td rowspan="4">5 µmol/L o14PEGMA(1:1:2.5)_NH_3</td><td rowspan="2">1.5 mmol/L PO_4^{3-}</td><td>Method 1</td>
<td>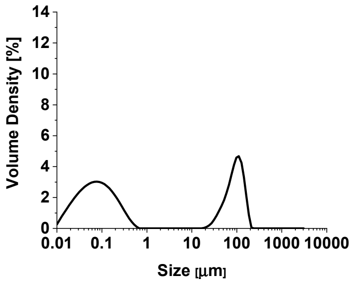

D[4;3] = 39.6 µm</td>
<td>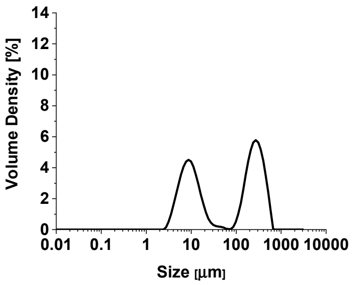

D[4;3] = 166 µm</td></tr>
<tr><td>Method 2</td>
<td>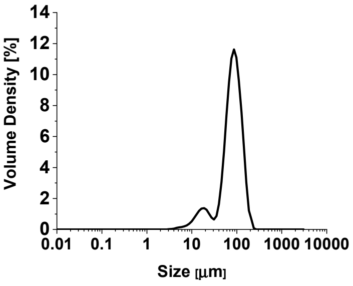

D[4;3] = 89.2 µm</td>
<td>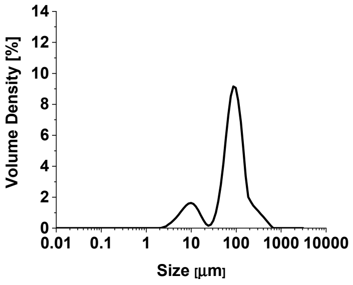

D[4;3] = 104 µm</td></tr>
<tr><td rowspan="2">6.0 mmol/L PO_4^{3-}</td><td>Method 1</td>
<td>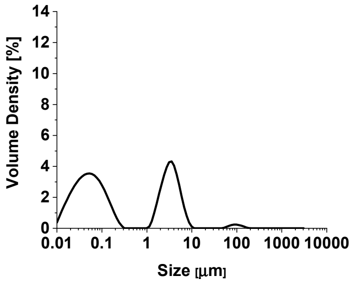

D[4;3] = 3.21 µm</td>
<td>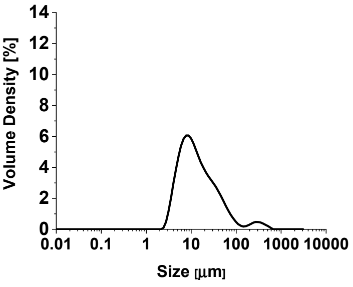

D[4;3] = 31.4 µm</td></tr>
<tr><td>Method 2</td>
<td>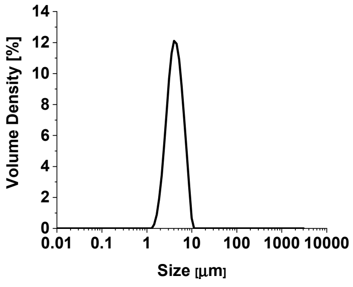

D[4;3] = 4.77 µm</td>
<td>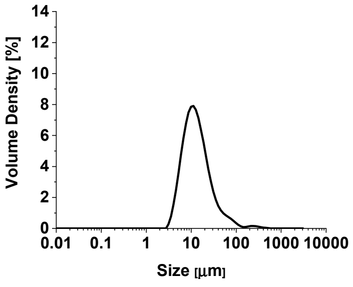

D[4;3] = 20.6 µm</td></tr>
</table>

			Size distribution	
			**t = 5 min**	**t = 30 min**
10 µmol/L o14PEGMA(1:1:2.5)_NH_3	1.5 mmol/L PO_4^{3-}	Method 1	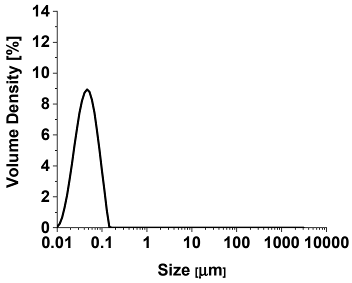 D[4;3] = 0.0535 µm	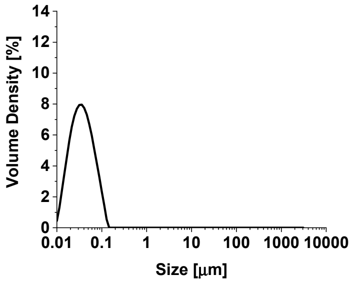 D[4;3] = 0.0442 µm
		Method 2	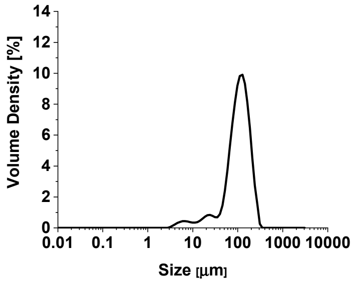 D[4;3] = 122 µm	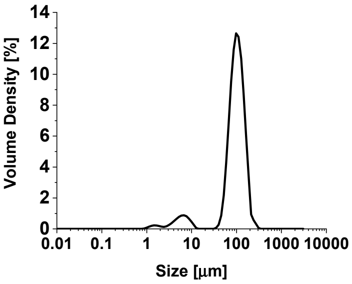 D[4;3] = 107 µm
	6.0 mmol/L PO_4^{3-}	Method 1	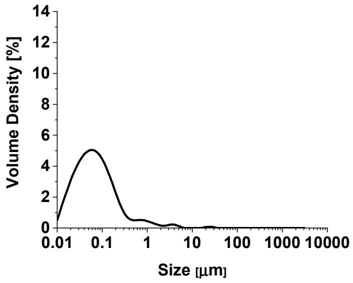 D[4;3] = 0.269 µm	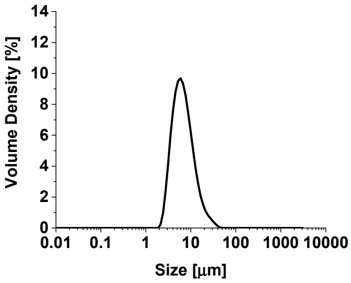 D[4;3] = 8.29 µm
		Method 2	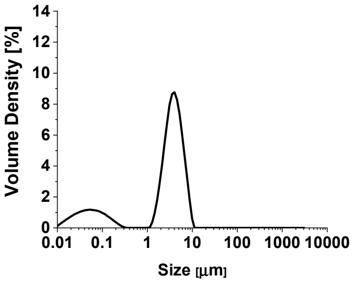 D[4;3] = 3.55 µm	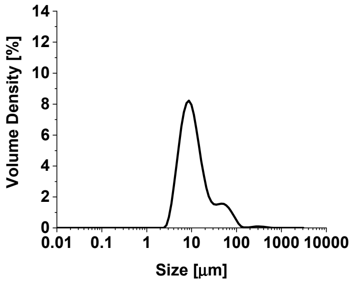 D[4;3] = 19.9 µm

Figure S3. Influence of oligomer concentration on size distribution and aggregation of CaP particles determined by Laser diffraction analysis.

NTA screenshots for analysis of aggregation status

			0 μmol/L siRNA	1 μmol/L siRNA	10 μmol/L siRNA
10 μmol/L o14PEGMA(1:1:2.5)_NH$_3$	1.5 mmol/L PO_4^{3-}	Method 1	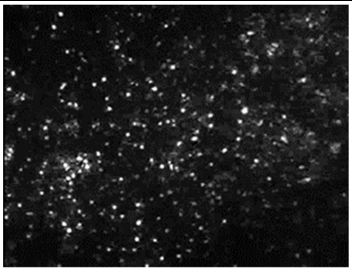	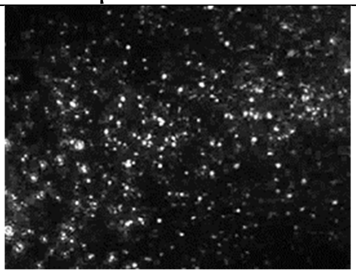	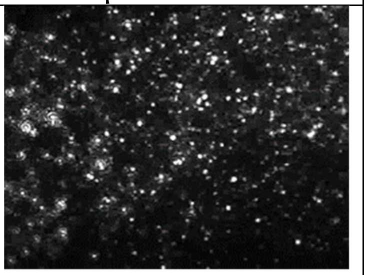
		Method 2	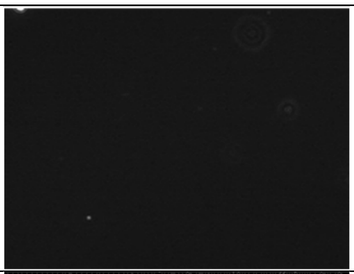	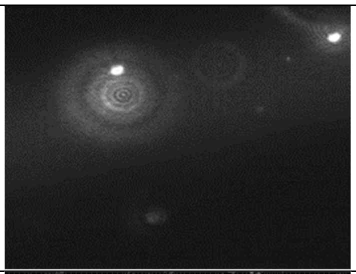	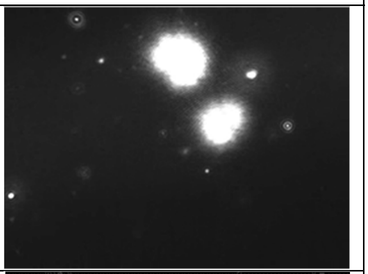
	3.75 mmol/L PO_4^{3-}	Method 1	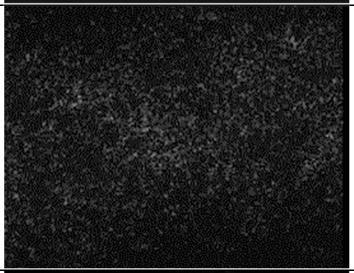	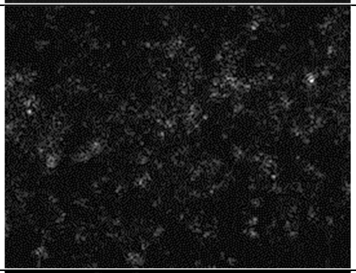	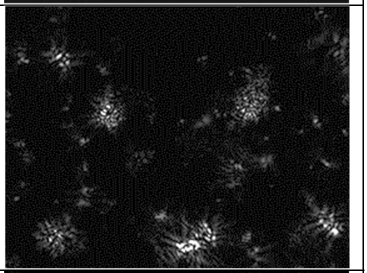
		Method 2	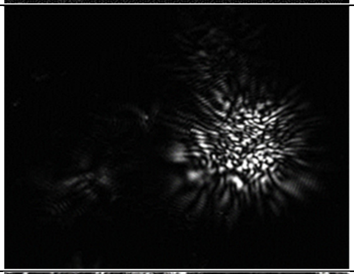	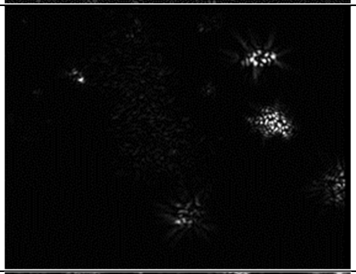	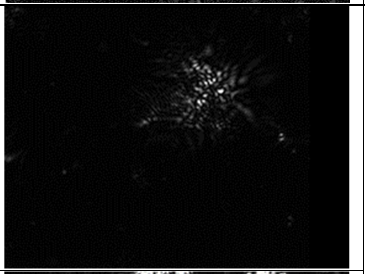
	6.0 mmol/L PO_4^{3-}	Method 1	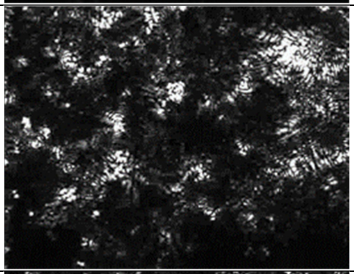	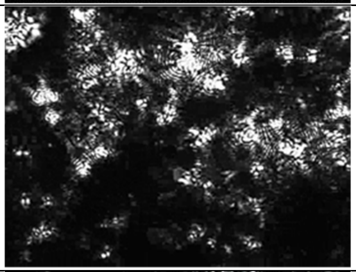	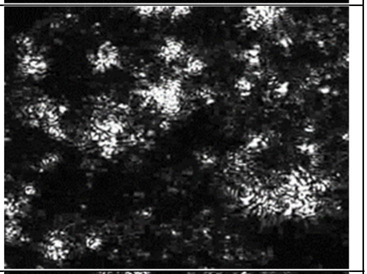
		Method 2	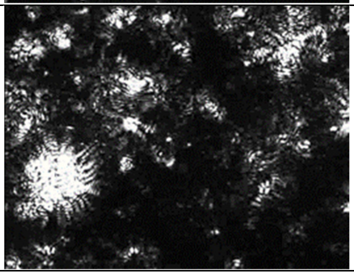	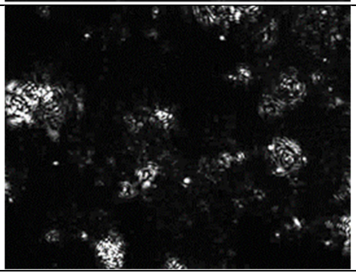	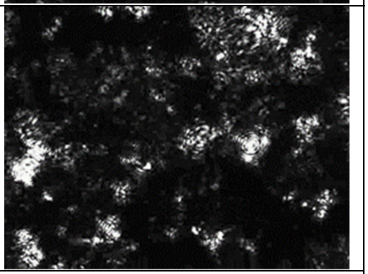
			0 μmol/L siRNA	1 μmol/L siRNA	10 μmol/L iRNA

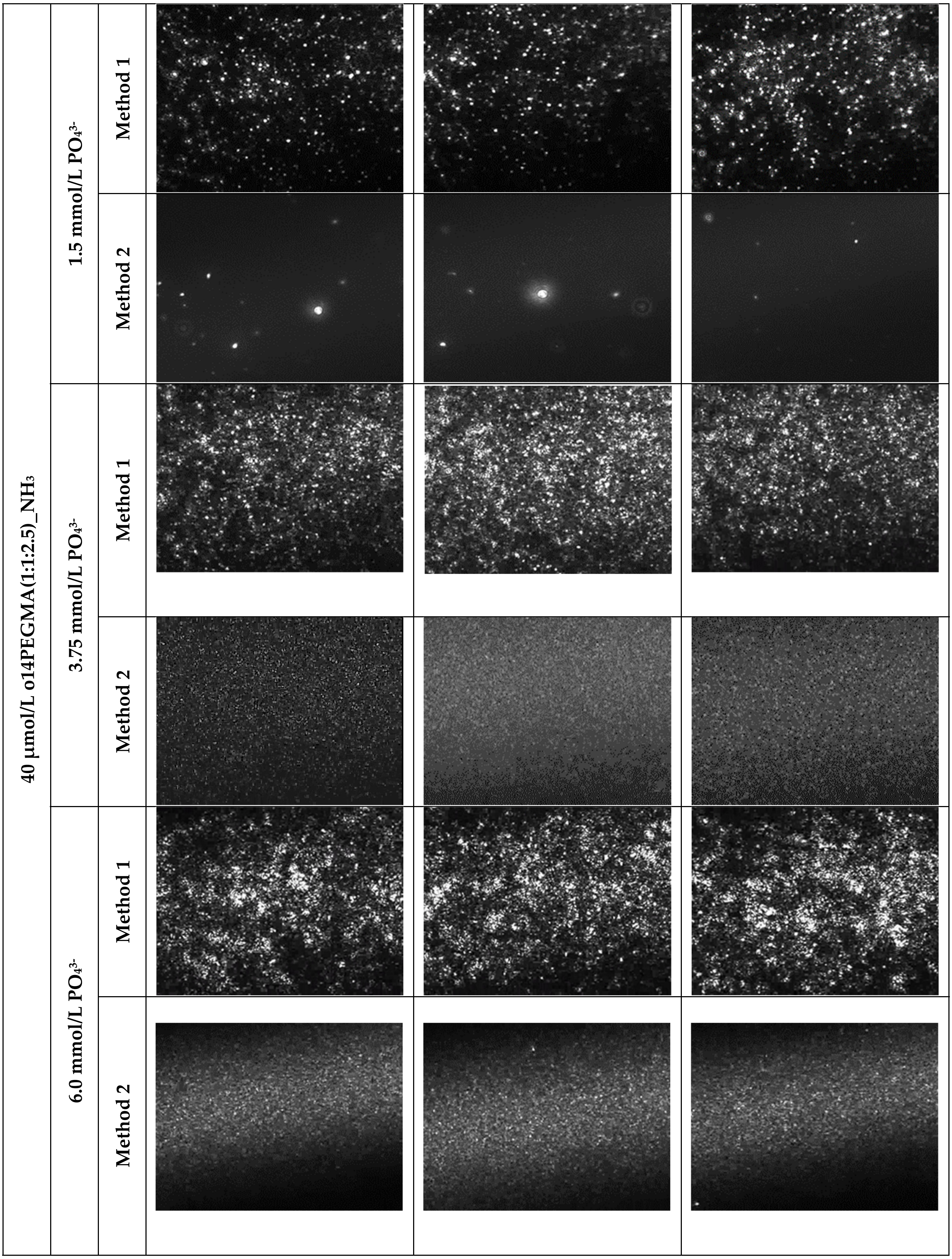

Figure S4. NTA screenshots of CaP-NP formulation 30 min after fabrication.

Impact of cell culture medium & serum proteins on size distribution of CaP-NP

		0 µmol/L siRNA	1 µmol/L siRNA
Oligomer/PO_4^{3-}−concentration/Method	40 µmol/L/3.75 mmol/L PO_4^{3-}/2	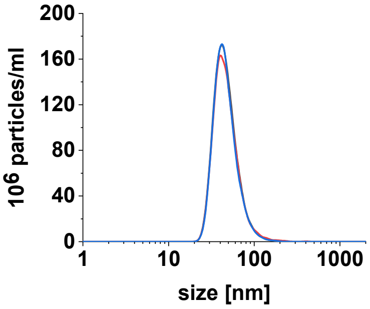<br>Mean: 55 ± 22 nm<br>Concentration: 57.8 ± 1.1*10⁸/ml<br>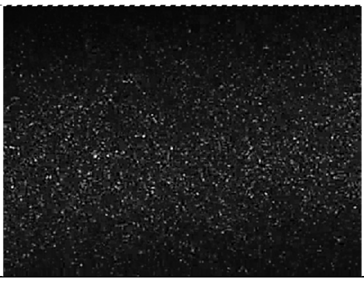	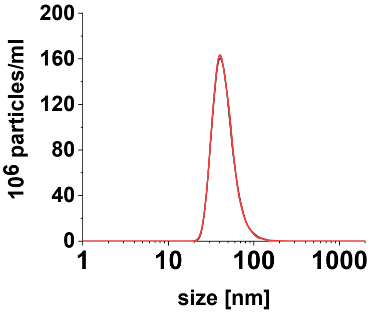<br>Mean: 52 ± 19 nm<br>Concentration: 49.7 ± 0.2*10⁸/ml<br>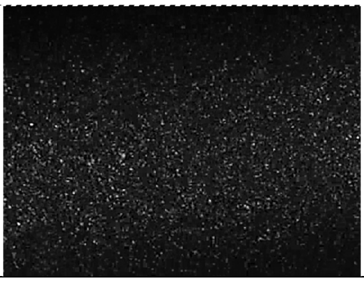
	40 µmol/L/6.0 mmol/L PO_4^{3-}/2	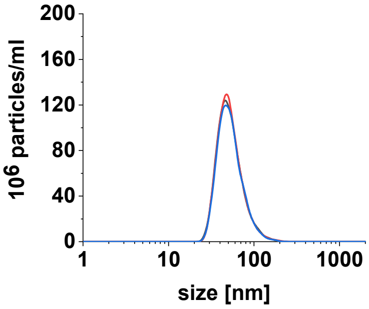<br>Mean: 63 ± 27 nm<br>Concentration: 51.8 ± 1.0*10⁸/ml<br>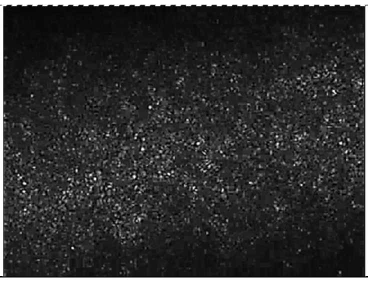	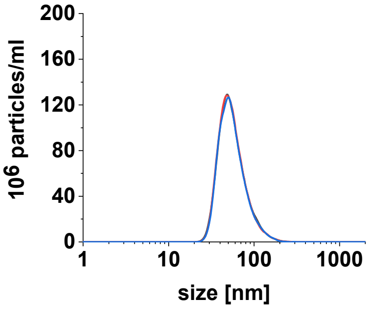<br>Mean: 69 ± 32 nm<br>Concentration: 58.6 ± 0.9*10⁸/ml<br>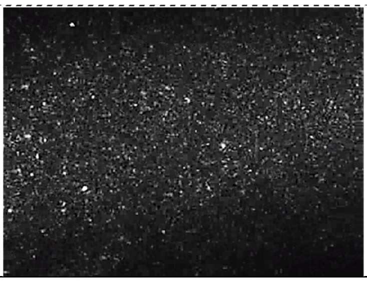

Figure S5. Effect of serum proteins and cell culture medium on stability of o14PEGMA(1:1:2.5)_NH_3-stabilized CaP-NP after 5 h.

Cellular Uptake of CaP-NP in F98 cells

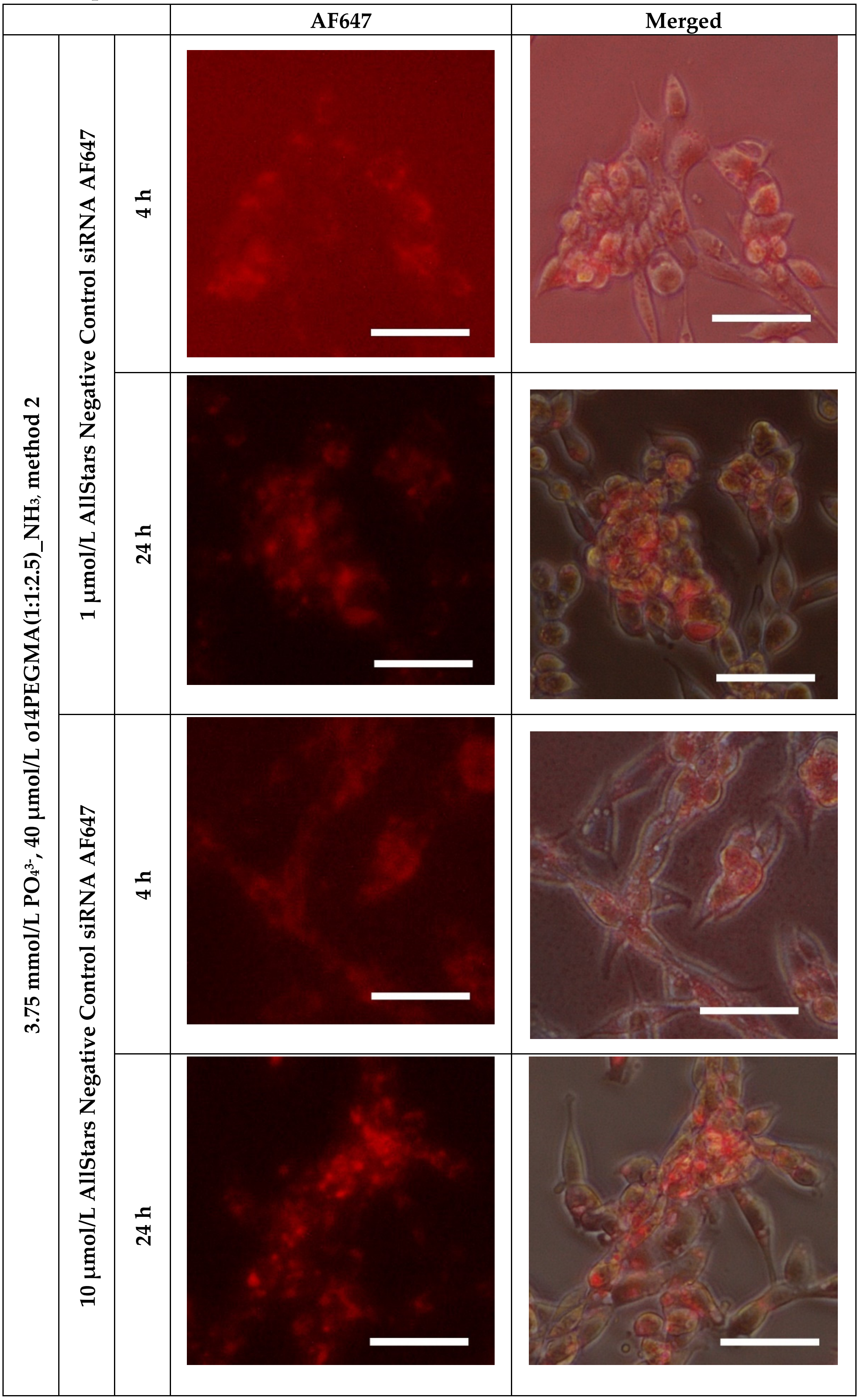

Figure S6. Uptake of siRNA (AlexaFluor™ 647 labeled)-loaded o14PEGMA(1:1:2.5)_NH_3-stabilized CaP-NP in F98 cells. Scale bars represent 100 µm.

Live/Dead staining

	Group		24 h	72 h
Non-concentrated	Control	Untreated	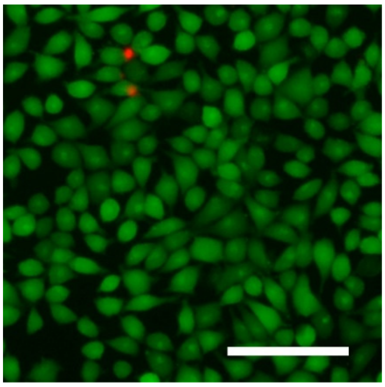	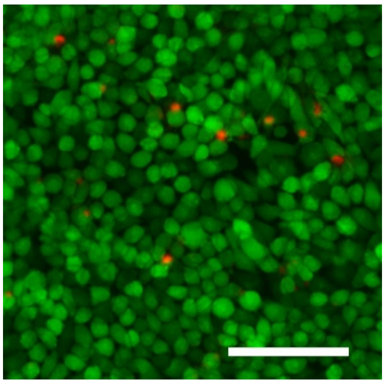
	3.75 mmol/L PO_4^{3-}, 40 µM o14PEGMA(1:1:2.5)_NH_3, method 2	0 µmol/L siRNA	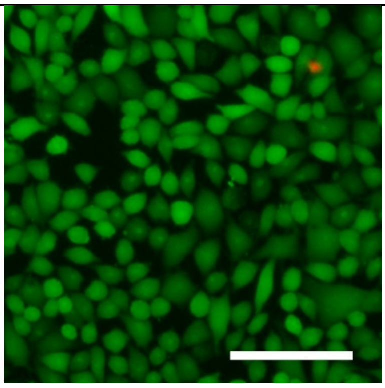	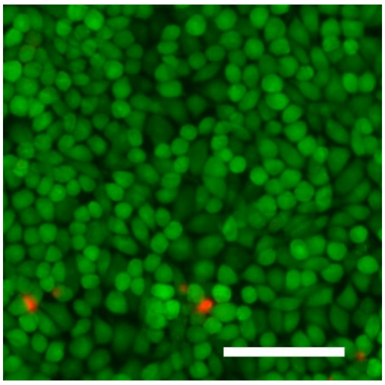
		1 µmol/L siRNA	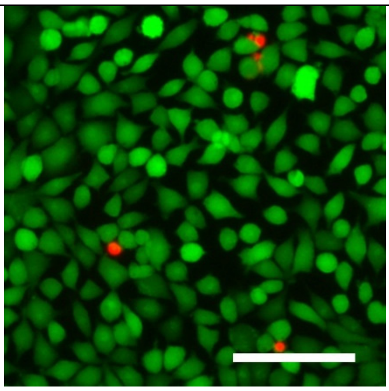	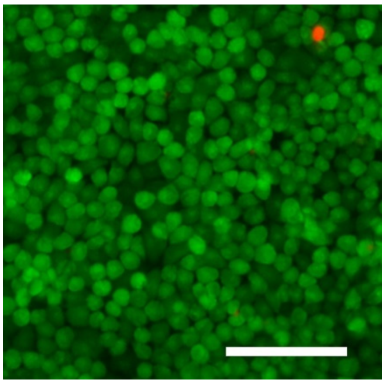

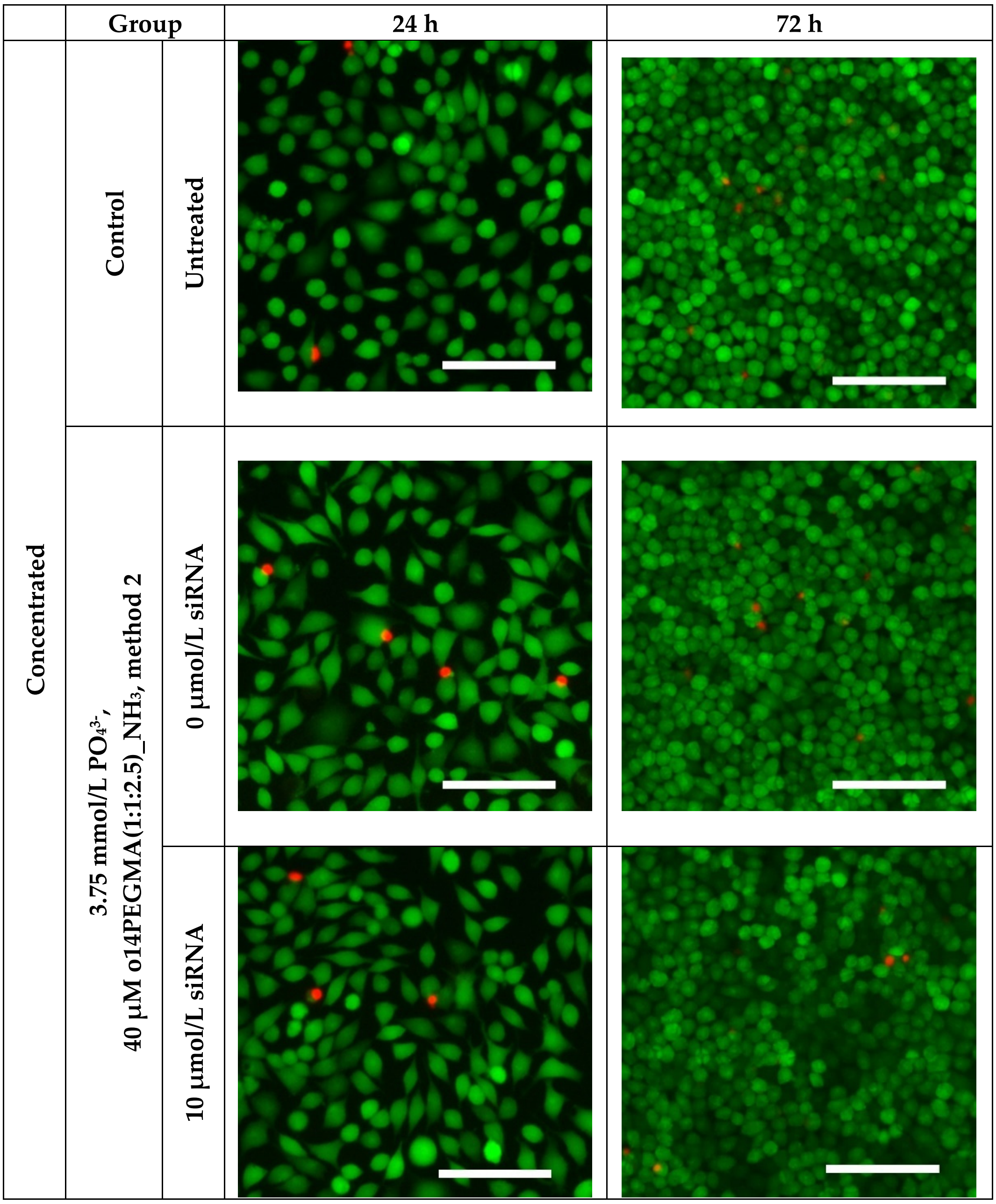

Figure S7. *In vitro* biocompatibility testing of CaP-NP in L929 cells: Live/Dead staining. Scale bars represent 100 µm.

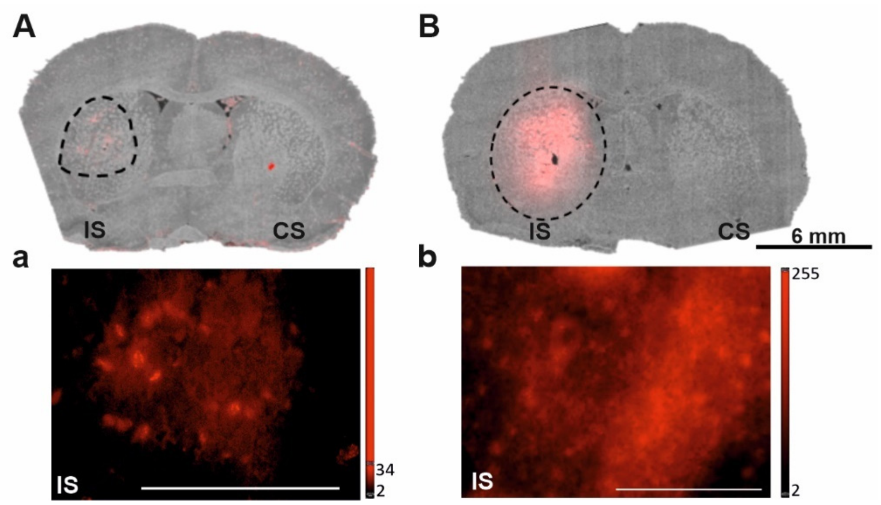

Figure S8. *In vivo* biodistribution of CaP-NP loaded with 10 µmol/L AllStars Negative Control siRNA AF647 (A, a) or AllStars Negative Control siRNA AF647 alone (B, b) 1 hour post-CED in healthy brain (n=2). Representative brain sections of fluorescence distribution in the whole slide (A, B) and fluorescence of the AllStars Negative Control siRNA AF647 at the injection site within the CaP-NP (a) and alone (b). IS: ipsilateral side (CED injection), CS: contralateral side (control) (scale bars represent 150 µm, x40 magnification).
